# A combined model of shoot phosphorus uptake based on sparse data and active learning algorithm

**DOI:** 10.3389/fpls.2024.1470719

**Published:** 2025-01-22

**Authors:** Tianli Wang, Yi Zhang, Haiyan Liu, Fei Li, Dayong Guo, Ning Cao, Yubin Zhang

**Affiliations:** ^1^ College of Plant Science, Jilin University, Changchun, China; ^2^ Agricultural College, Henan University of Science and Technology, Luoyang, Henan, China; ^3^ College of Grassland, Resources and Environment, Inner Mongolia Agricultural University, Hohhot, China

**Keywords:** 2DCOS, 2T2DCOS, active learning, phosphorus uptake, PROSAIL-5B

## Abstract

The soil ecosystem has been severely damaged because of the increasingly severe environmental problems caused by excessive application of phosphorus (P) fertilizer, which seriously hinders soil fertility restoration and sustainable farmland development. Shoot P uptake (SPU) is an important parameter for monitoring crop growth and health and for improving field nutrition management and fertilization strategies. Achieving on-site measurement of large-scale data is difficult, and effective nondestructive prediction methods are lacking. Improving spatiotemporal SPU estimation at the regional scale still poses challenges. In this study, we proposed a combination prediction model based on some representative samples. Furthermore, using the experimental area of Henan Province, as an example, we explored the potential of the hyperspectral prediction of maize SPU at the canopy scale. The combination model comprises predicted P uptake by maize leaves, stems, and grains. Results show that (1) the prediction accuracy of the combined prediction model has been greatly improved compared with simple empirical prediction models, with accuracy test results of *R* ^2^ = 0.87, root mean square error = 2.39 kg/ha, and relative percentage difference = 2.71. (2) In performance tests with different sample sizes, two-dimensional correlation spectroscopy i.e., first-order differentially enhanced two-dimensional correlation spectroscopy (1Der-2DCOS) and two-trace 2DCOS of enhanced filling and milk stages (filling-milk-2T2DCOS)) can effectively and robustly extract spectral trait relationships, with good robustness, and can achieve efficient prediction based on small samples. (3) The hybrid model constrained by the Newton-Raphson-based optimizer’s active learning method can effectively filter localized simulation data and achieve localization of simulation data in different regions when solving practical problems, improving the hybrid model’s prediction accuracy. The practice has shown that with a small number of representative samples, this method can fully utilize remote sensing technology to predict SPU, providing an evaluation tool for the sustainable use of agricultural P. Therefore, this method has good application prospects and is expected to become an important means of monitoring global soil P surplus, promoting sustainable agricultural development.

## 1 Introduction

One important means of increasing crop yield and ensuring global food security is the rational application of phosphorus (P) fertilizer (Mogollón et al., 2021; Langhans et al., 2022; McDowell et al., 2024). However, in the past four decades, the application of P fertilizer has exceeded the global sustainable development’s “planetary boundary” because of the increasing demand for P in agricultural production (Steffen et al., 2015; Springmann et al., 2018). The excessive application of P fertilizer in global farmland increases agricultural production costs (Hu et al., 2020), intensifies the crisis of P resources (Cooper et al., 2011), and causes nutritional pollution because of the excessive leaching of P into adjacent water bodies (Zou et al., 2022). This poses a severe threat to the water environment, which seriously hinders the achievement of sustainable development goals. Therefore, there is an urgent need for large-scale, long-term, and reliable monitoring of shoot P uptake (SPU) to meet the growing demand for food (Mueller et al., 2012) and address the dual challenges of intensified P pollution and reduced P reserves (Mew, 2016).

Many studies have been conducted on estimating SPU, and three commonly used methods exist. First, some literature multiply statistical yearbook data at the regional scale with crop nutrient concentrations to obtain nutrient uptake (Yin et al., 2021; Zou et al., 2022). Although this method is easier to implement on a regional scale, the data in statistical yearbooks usually have substantial spatial errors and may have substantial statistical errors with measurement data, resulting in low accuracy. Second, some literature directly uses nutrient uptake efficiency to calculate nutrient uptake (Jiao et al., 2015; Zhu et al., 2022). Third, multiply the fixed nutrient concentration with biomass to obtain the nutrient uptake (Wen et al., 2017). Although these two methods are simple, they lack flexibility, require collecting a large number of ground measurement samples to compensate for the loss of accuracy, and have poor universality because nutrient uptake efficiency and concentration are often variable in different environments, species, or regions (He et al., 2020). Distinct sampling methods based on limited sample sizes are insufficient to monitor SPU spatial distribution (Marshall and Thenkabail, 2015). It is difficult to achieve large-scale routine field investigations because of time and manpower limitations (Mahajan et al., 2017; Abdel-Rahman et al., 2017; Wu et al., 2022). Recently, various crop canopy information with spatial full coverage and temporal continuity can be obtained (Gracia-Romero et al., 2017; Gao et al., 2019; Belgiu et al., 2023) because of the development of hyperspectral remote sensing technology (Pang et al., 2022), providing the possibility for predicting SPU.

Previous studies have mostly focused on leaf P concentration (LPC) (Pandey et al., 2017; Ge et al., 2019), Aboveground biomass except for grains (AGB) (Zhang et al., 2021b; Wocher et al., 2022), and grain yield (Yang et al., 2021; Yue et al., 2023) in maize. There has been relatively little quantitative analysis of P uptake in maize aboveground tissues (i.e., leaves, stems, and grains) under field conditions. However, problems still exist even if the predictive models for these three factors are relatively mature. First, the extracted features lack good representativeness (Pandey et al., 2017; Yang et al., 2021; Zhang et al., 2021b), resulting in a lack of universality in the wavelength selection of prediction models under different environments and conditions. Second, for feature selection methods, the contradiction between the computational intensity and accuracy of optimal band selection is always an issue that cannot be ignored (Yang et al., 2021; Sun et al., 2021). To address the aforementioned issues, we must strongly conduct sufficient data mining with less computational complexity on hyperspectral datasets to extract complete sensitive bands. In addition, there was a strong correlation between AGB and canopy spectra at different growth stages. Considering hyperspectral data at different growth stages, two-trace two-dimensional correlation spectroscopy (2T2DCOS) (Noda, 2018, 2022) will provide the potential to extract important spectral information by fully exploring the information contained in spectra at different growth stages.

Moreover, because of the occlusion of crop canopies, particularly under dense canopy conditions such as corn, optical sensors cannot receive most of the spectral information of the obstructed organs (Li et al., 2023), making it difficult to directly predict P concentration and P uptake at the canopy scale. Therefore, exploring the uptake relationship of P among different organs (e.g., corn leaves, stems, and grains) has high scientific significance and practical value. Thus, this study’s focus and challenge is overcoming these limiting factors and improve the accuracy and reliability of hyperspectral data to predict crop SPU. There are currently three main prediction models: data-driven methods (Atzberger et al., 2015), physical methods (Féret et al., 2017), and hybrid methods (Kayad et al., 2022). Data-driven methods are relatively easy to implement but cannot meet large-scale monitoring requirements. Physical model requires a large number of input parameters (Atzberger et al., 2010), and the model structure is complex; therefore, the application of this method is still quite limited. Thus, among them, hybrid models are increasingly favored by researchers. The hybrid method combines the advantages of these two methods (Verrelst et al., 2019), alleviating the limitations of data-driven and physical models (Berger et al., 2020a). However, the performance of hybrid methods is limited by the quality of the simulated datasets during the training process. If there is a substantial difference between the simulated and measured datasets, it may not be possible to effectively quantify leaf biomass from canopy reflectance. Previous studies have attempted to update simulated datasets using real ground samples and successfully applied hybrid methods to real-world scenarios (Féret et al., 2019). However, they mainly used random selection to obtain new samples, which may not be representative and may even harm model performance (Wan et al., 2023). Compared with random selection, active learning methods can select representative samples (Berger et al., 2020b) and establish robust models with small datasets (Verrelst et al., 2016), which will be beneficial for developing effective hybrid methods for estimating leaf biomass. Furthermore, SPU combination prediction models suitable for different environments, conditions, and scales can be developed by combining empirical models to provide more accurate and reliable decision support for agricultural production and promote sustainable agricultural development.

In this study, we used the Gaolong Town Experimental Zone in Yanshi District, Luoyang City, Henan Province, as an example. Moreover, we used two-dimensional correlation spectra and active learning algorithms to construct a combination model to address the following core issues: (i) explore the effectiveness and robustness of extracting spectral trait relationships from two-dimensional correlation spectra, with the expectation of establishing robust prediction models based on a small number of representative samples; (ii) explore the effectiveness of using active learning to optimize simulated data, with the expectation of obtaining more localized training data; (iii) explore how to use canopy reflectance spectroscopy to achieve nondestructive prediction of P uptake in maize stems and grains; and (iv) establish and evaluate corn SPU combination prediction models. By predicting maize SPU based on remote sensing data, we can provide a basis for studying soil P surplus, guiding local fertilization strategies, and providing a scientific basis for improving P utilization efficiency and achieving sustainable agricultural development.

## 2 Materials and methods

### 2.1 Research material

#### 2.1.1 Study area and experimental design

The research area is located in Gaolong Town, Yanshi District, Luoyang City, Henan Province (112°41′49″E, 34°36′6″). The annual average temperature and precipitation are 14.2°C and 579.7 mm, respectively. Annual precipitation’s spatial and temporal distribution is uneven, with rainfall concentrated from June to July each year. The basic physicochemical properties of the experimental area are as follows: soil pH, organic matter, total N, total P, available N, and available P were 7.8, 17.1 g·kg^-1^, 0.67 g·kg^-1^, 0.31 g·kg^-1^, 29.8 mg·kg^-1^, and 8.9 mg·kg^-1^, respectively. We selected the Zhengdan 958 variety, where continuous positioning fertilization treatment has been implemented for many years. The experimental area was designed as a randomized block experiment, with a size of 10 m × 7.2 m. Nitrogen fertilizer (urea, N 45%) and potassium fertilizer (potassium chloride, K_2_O 50%) were applied as base fertilizers at 90 and 60 kg/ha, respectively.

**Figure 1:**
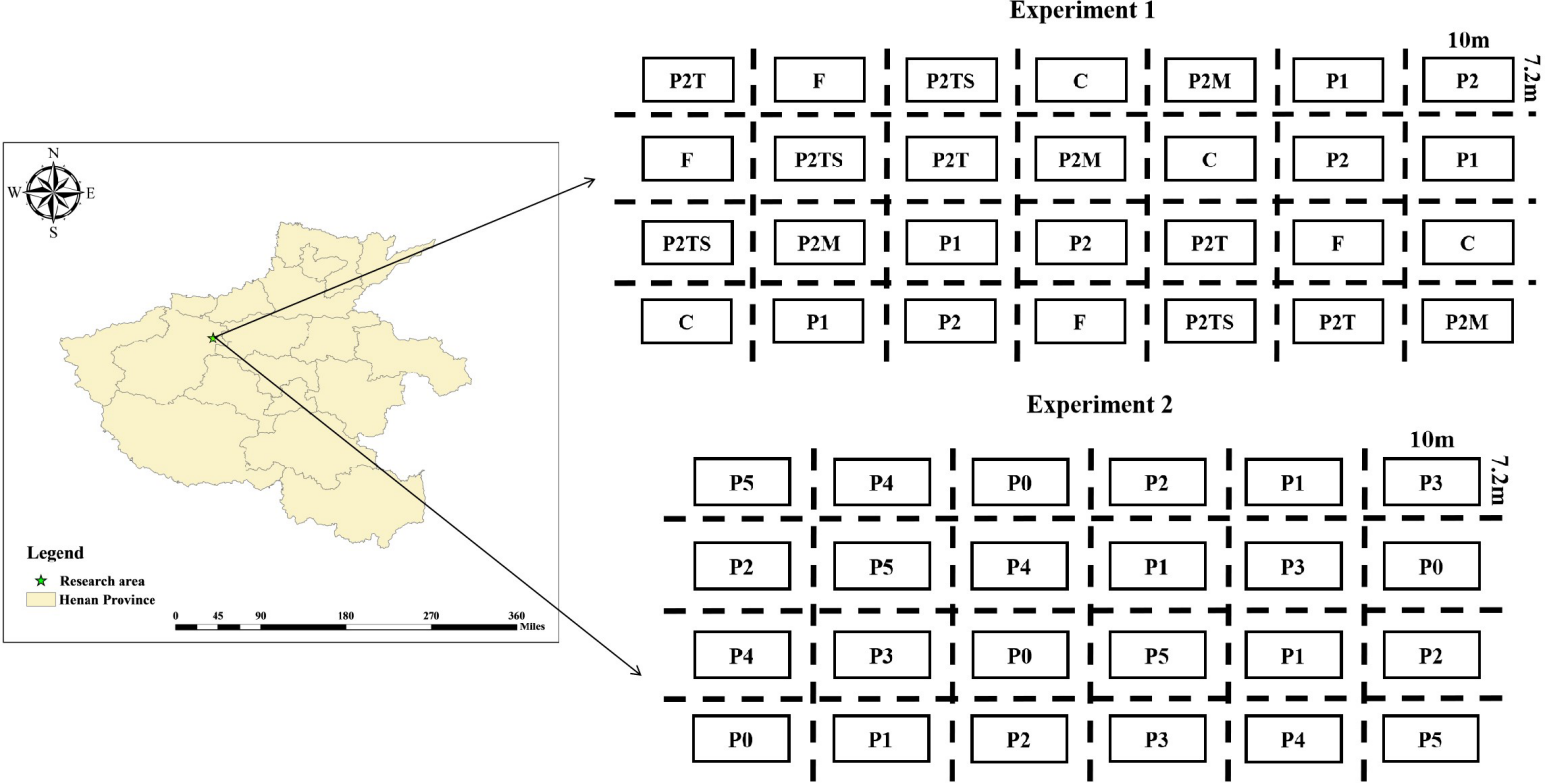
Experimental setup in the research area.

As shown in **Figure 1**, we set up experiment 1 with seven P treatments using calcium superphosphate (P_2_O_5_ 12%) and polyphosphate (H_12_N_3_O_4_P 40%) as P fertilizers, with P application rates of F: 90 kg (P_2_O_5_)/ha; C: 65 kg (P_2_O_5_)/ha; P1: 45 kg (P_2_O_5_)/ha; P2: 32.5 kg (P_2_O_5_)/ha; P2T: 9 kg (P_2_O_5_)/ha; P2TS: 9 kg (P_2_O_5_)/ha; and P2M: 9 kg (H_12_N_3_O_4_P)/ha. We performed four replicates per treatment. Urea (N 45%) was applied at 135 g/ha during the six-leaf stage, and P2T, P2TS, and P2M treatments added 23.5 kg/ha of calcium superphosphate (P_2_O_5_ 12%).

We set up experiment 2 with six P treatments using calcium superphosphate (P_2_O_5_ 12%) as the P fertilizer. The P application treatments were P0: 0 kg (P_2_O_5_)/ha; P1: 15 kg (P_2_O_5_)/ha; P2: 30 kg (P_2_O_5_)/ha; P3: 60 kg (P_2_O_5_)/ha; P4: 90 kg (P_2_O_5_)/ha; and P5: 120 kg (P_2_O_5_)/ha. We repeated this procedure four times for each treatment.

#### 2.1.2 Field observation

To measure the leaves of different layers during the jointing, filling, and milk stages of summer maize in 2021, we used a PSR+3500 spectrophotometer (Spectral Evolution Inc., Lawrence, MA, USA). We selected three representative samples from each experimental area, with upper, middle, and lower leaves taken from three different layers. We also selected two leaves from each layer for measurement, and a standard reference whiteboard was used for calibration before measurement. The blade is equipped with an active light source while ensuring its stability. The handle blade should be clamped in the middle of the blade, avoiding the leaf vein. At the same time, the blade clamp should be kept perpendicular to the blade. Finally, the spectral data should be obtained and stored in PAD. We took the leaves measured in the experimental area back to the laboratory for drying, where they were ground into powder using a grinder. Finally, to determine the P concentration in corn leaves, we used the sulfuric acid hydrogen peroxide digestion vanadium molybdenum yellow colorimetric method. We used the upper and middle leaves during the jointing stage and the upper, middle, and lower leaves during the filling stage as mature maize leaves. To simulate the P concentration in the plant canopy, we calculated the average P concentration in different leaf layers.

At the end of the growth cycle, we measured the biomass and P concentration of different aboveground maize tissues in experiment 1. We also measured AGB of maize in experiment 2.

### 2.2 Model modification

In real-world application scenarios, training an effective prediction model relies on a large number of measured samples, whereas accurately measuring large-scale data is often time-consuming, labor-intensive, and costly. Active learning is one of the main ways to reduce the cost of sample collection. Therefore, we applied an active learning method, namely the sampling strategy of NRBO-AL. We iteratively selected the most valuable simulation data and enhanced the generalization ability of the training model based on the simulation dataset to actual scenarios to improve the performance of the prediction model with minimum cost.

The specific implementation steps are as follows.

1. We used the measured samples as the measurement dataset $X_{n},n=1,\cdots ,N$ . By considering representativeness, we selected *K*  (K<N) measurement datasets as representative samples $X_{m},m=1,\cdots ,K$ .
2. Based on the NRBO algorithm (Sowmya et al., 2024) (see **Appendix S2.2** for details), we obtained a simulated dataset $X_{t},t=1,\cdots ,T$ , that is closer to the centroid of representative samples.

The shortest distance from $X_{t}$ to $X_{m}$ is represented as follows:


(1) $$d_{t}^{x}=||x_{t}-x_{m}||<\epsilon ,\;x_{t}\in X_{t},\;x_{m}\in X_{m}$$

3. We established a multiple linear regression model *f*(*x*) based on representative samples and selected simulated data samples with a smaller distance from the simulated biomass $y_{t}$ as the updated training dataset. We also used the updated training dataset to train regression models for estimating biomass in the measured dataset.

Among them, the distance from $f\left(x_{t}\right)$ to $y_{t}$ is represented as follows:


(2) $$d_{t}^{x}=||f\left(x_{t}\right)-y_{t}||<\epsilon ,x_{t}\in X_{t}$$

We selected a new sample from the measurement dataset for each iteration to update the training dataset until the maximum number of samples (*K*) was reached. To balance sampling costs and model performance, we usually set the maximum number of new samples to 10% of the measured datasets (Wan et al., 2020, 2022). However, for small datasets, *K* will be set higher (Wan et al., 2023), and if too few samples are selected, effective features cannot be learned.

The traditional random selection method is based on the maximum number of new samples, and it directly selects samples from the measurement dataset, which cannot guarantee the quality of new samples. Contrary to this random method, the greedy algorithm based on NRBO can impose constraints on the quality of new samples, thereby enhancing the potential to improve model performance beyond random selection.

### 2.3 Technical approach

**Figure 2:**
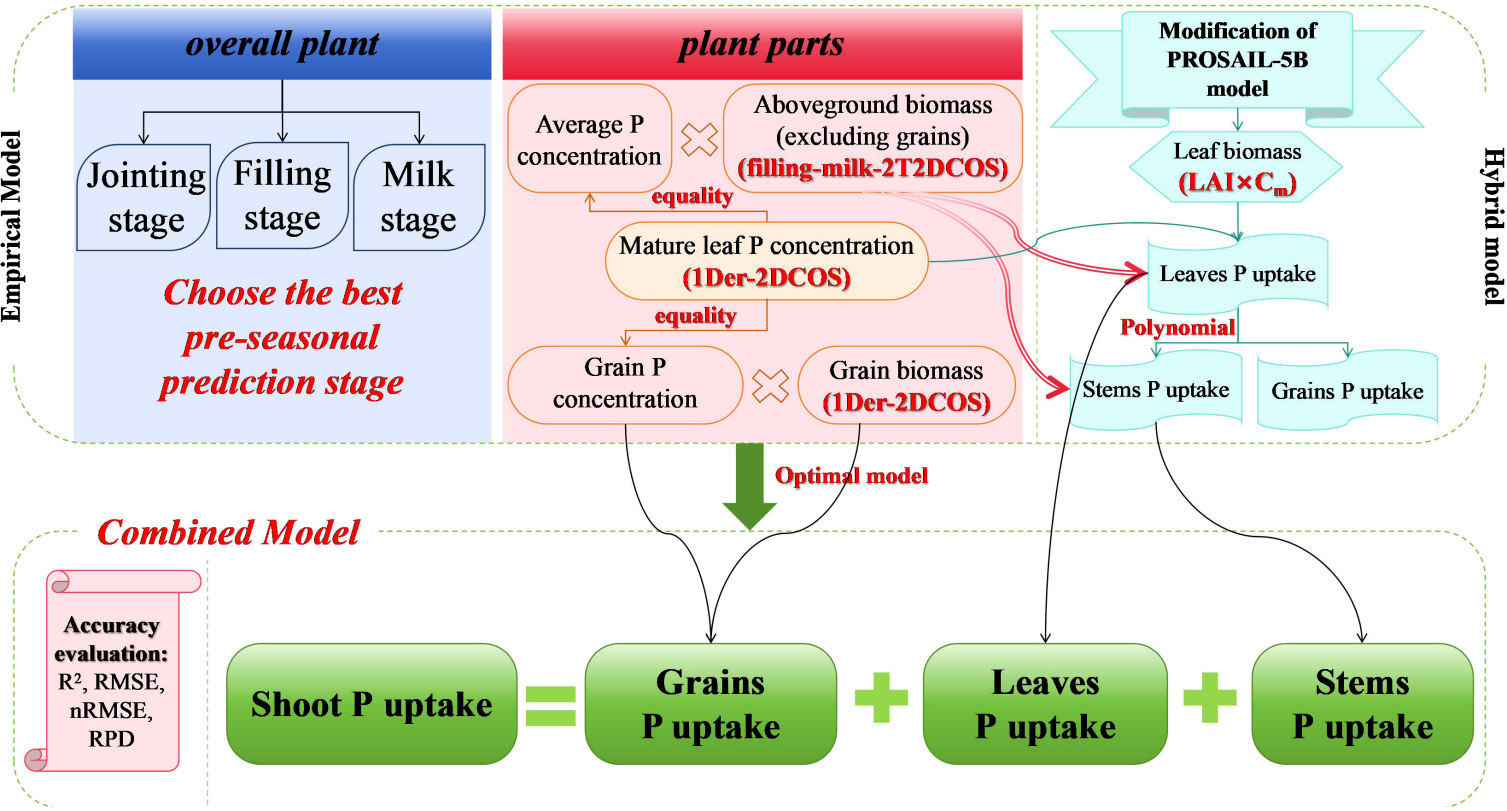
Schematic representation of the technical approach.

To achieve high-precision prediction mechanisms for maize SPU, we comprehensively explored and analyzed the use of small-scale remote sensing data (**Figure 2**). We established two empirical models based solely on actual ground measurement data based on two-dimensional correlation spectra and a hybrid model based on Newton–Raphson-based optimizer’s active learning (NRBO-AL) optimization and a combined prediction model combining mixed models and partial empirical models.

We used 2T2DCOS (see **Appendix S2.3.1**) to couple the spectra of the filling and milk stages to establish an empirical model. We selected new spectral features based on peak position screening for accurate AGB prediction. We also used first-order differentially enhanced two-dimensional correlation spectroscopy (1Der-2DCOS) (see **Appendix S2.3.2**) to analyze the spectral changes under P concentration disturbance, thereby predicting the P concentration in maize canopy leaves. We multiplied the two as SPU, except for the grains. For predicting P uptake by grains, we utilized 1Der-2DCOS to analyze the spectral changes under yield disturbances, thus establishing a prediction model for maize kernel biomass. We multiplied this by the P concentration in the seeds to determine the amount of P absorbed by the seeds.

To establish a hybrid model for predicting leaf biomass, we conducted a sensitivity analysis of the parameters in the PROSAIL-5B model (Jacquemoud et al., 2009) (see **Appendix S2.3.3**). We determined the parameter’s sensitivity through EFAST sensitivity analysis (Qiao et al., 2020) (see **Appendix S2.3.4**) and using the mixing sine and cosine algorithm with the Lévy flying chaotic sparrow algorithm (Qinghua et al., 2021) (see **Appendix S2.3.5**) to optimize the model parameters and determine the range of high-sensitivity parameters and the values of other low-sensitivity parameters. Subsequently, we screened the simulation dataset generated by the PROSAIL-5B model using the NRBO-AL method, which comprehensively improved the PROSAIL-5B model’s effectiveness, achieving localization of model parameters and high-precision prediction of leaf biomass. We multiplied this by the P concentration in the canopy leaves to determine the amount of P absorbed by corn leaves. Subsequently, we established a prediction model for P uptake by stems and grains based on P uptake by corn leaves.

Using actual observed data, we evaluated the accuracy of the four SPU combination prediction models by determining the coefficient (*R* ^2^), root mean square error (RMSE), and relative percentage difference (RPD) (see **Appendix S2.3.6**) and selecting the best prediction model from them.

## 3 Results

### 3.1 Stable prediction model for small-size datasets

Taking the prediction of whole reproductive period LPC as an example, we selected different sample sizes to investigate the possibility of stable prediction on small datasets. We selected samples consistent with the entire dataset’s distribution, namely 10%, 20%, 30%, 40%, 50%, 60%, 70%, 80%, 90%, and 100% samples (n=252), and compared and analyzed the stability of different dimensionality reduction methods and the combination effect of prediction methods.

We applied derivative transformation to the spectrum to eliminate background noise, and compared the stability of different dimensionality reduction methods such as Successive Projections Algorithm (SPA), Least Absolute Shrinkage and Selection Operator (LASSO), (Elastic Net) EN, and 2DCOS. As the number of repetitions decreased from 10 to 5, **Table 1** recorded the changes in the number of bands under different methods. The SPA method has almost no repeated band selection, while both the EN and 2DCOS methods have repeated band selection. However, at a repetition rate of 10, the 2DCOS method shows a higher number of bands. When the number of repetitions decreases, the number of bands in the EN method slightly increases, while the 2DCOS method shows a stable trend. This set of data reveals the dynamic variation characteristics of the number of bands under different methods as the number of repetitions changes. The selected bands for 2DCOS dimensionality reduction are independent of the amount of data, exhibiting strong stability and unique advantages (Yue et al., 2021).

**Table 1:** Each type of preprocessing method repeatedly selects the band number distribution of different datasets.

Repetitions	Number of bands								
	SPA			EN			2DCOS		
	Raw	1-Der	2-Der	Raw	1-Der	2-Der	Raw	1-Der	2-Der
10	/	/	/	4	1	1	8	110	229
9	/	/	/	5	1	2	6	86	118
8	/	/	/	11	2	2	4	44	88
7	/	/	/	34	3	5	2	44	70
6	/	/	/	53	5	12	14	40	64
5	1	1	/	77	14	16	14	32	48

**Figure 3:**
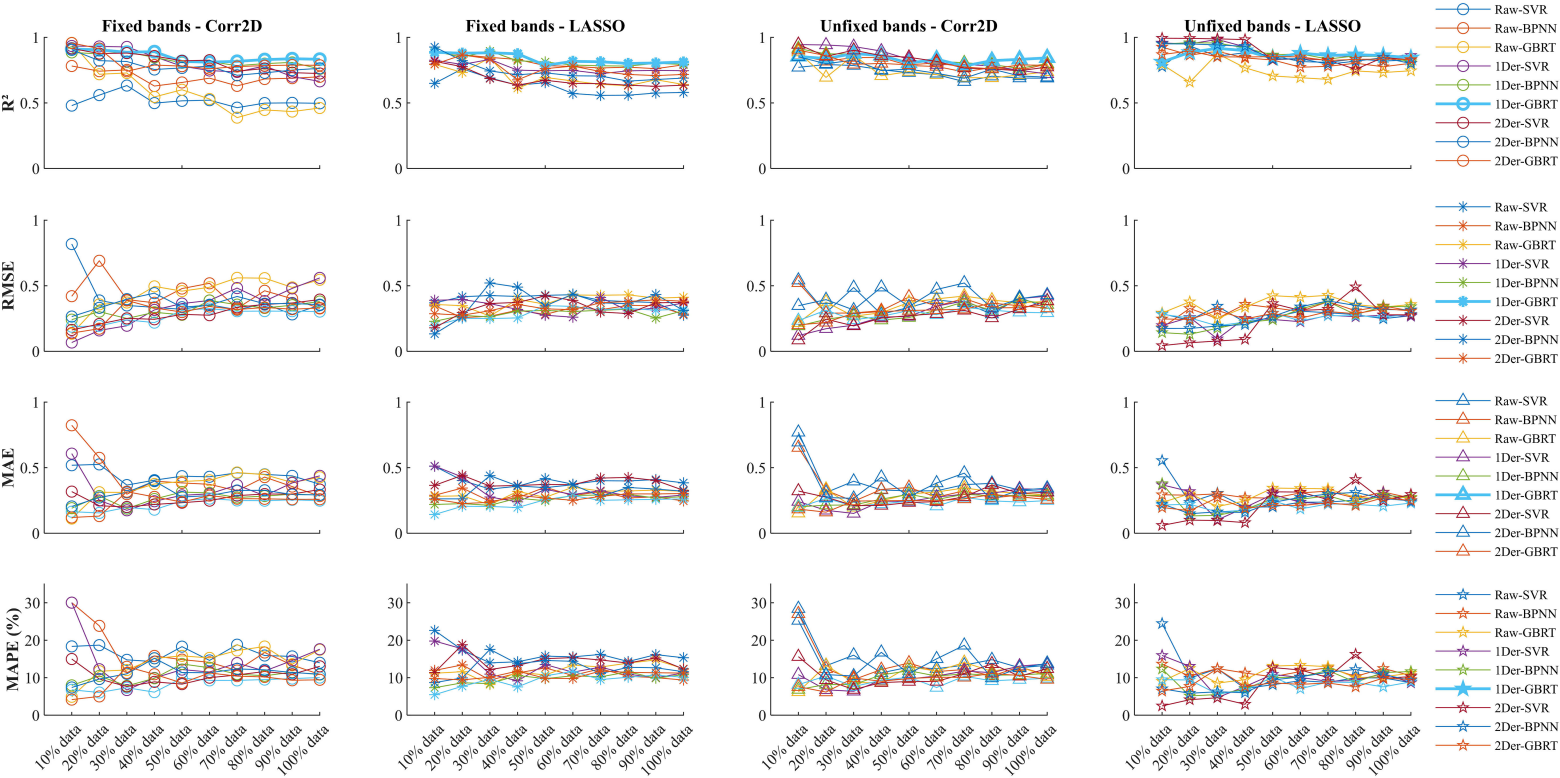
The prediction performance under different treatments.

In this study, we selected bands with more than 8 datasets that were repeatedly selected under 6 different preprocessing methods to test the LPC prediction performance. Among them, we compared the prediction performance of selecting different bands and the same band under different data partitioning (as shown in **Figure 3**).

From **Figure 3**, it can be observed that for the raw data, the prediction performance when selecting the same sensitive bands is inferior to when selecting different sensitive bands (-0.1275 [-0.1022, -0.1529]). This suggests that the sensitive bands in the raw data are unstable and susceptible to interference from external factors such as background noise, leading to shifts in the sensitive bands. Therefore, after removing the impact of background noise using derivative transformation, the prediction performance significantly improves. Under the first-order derivative preprocessing, for datasets with non-uniform bands, EN’s dimensionality reduction performs better than 2DCOS. This is because the sensitive bands after EN dimensionality reduction have lower redundancy, indicating that EN dimensionality reduction has poor universality and is more influenced by the heterogeneity of the dataset. In contrast, on a uniform dataset, 2DCOS shows better prediction performance because it has higher universality in dimensionality reduction, performing well across different datasets with minimal model tuning required. Additionally, under 2DCOS dimensionality reduction, regardless of whether fixed bands are selected, the prediction performance difference is very small (-0.0033 [-0.0119, 0.0052]). Therefore, combining derivative transformation with 2DCOS dimensionality reduction provides the most stable regression model.

**Figure 4:**
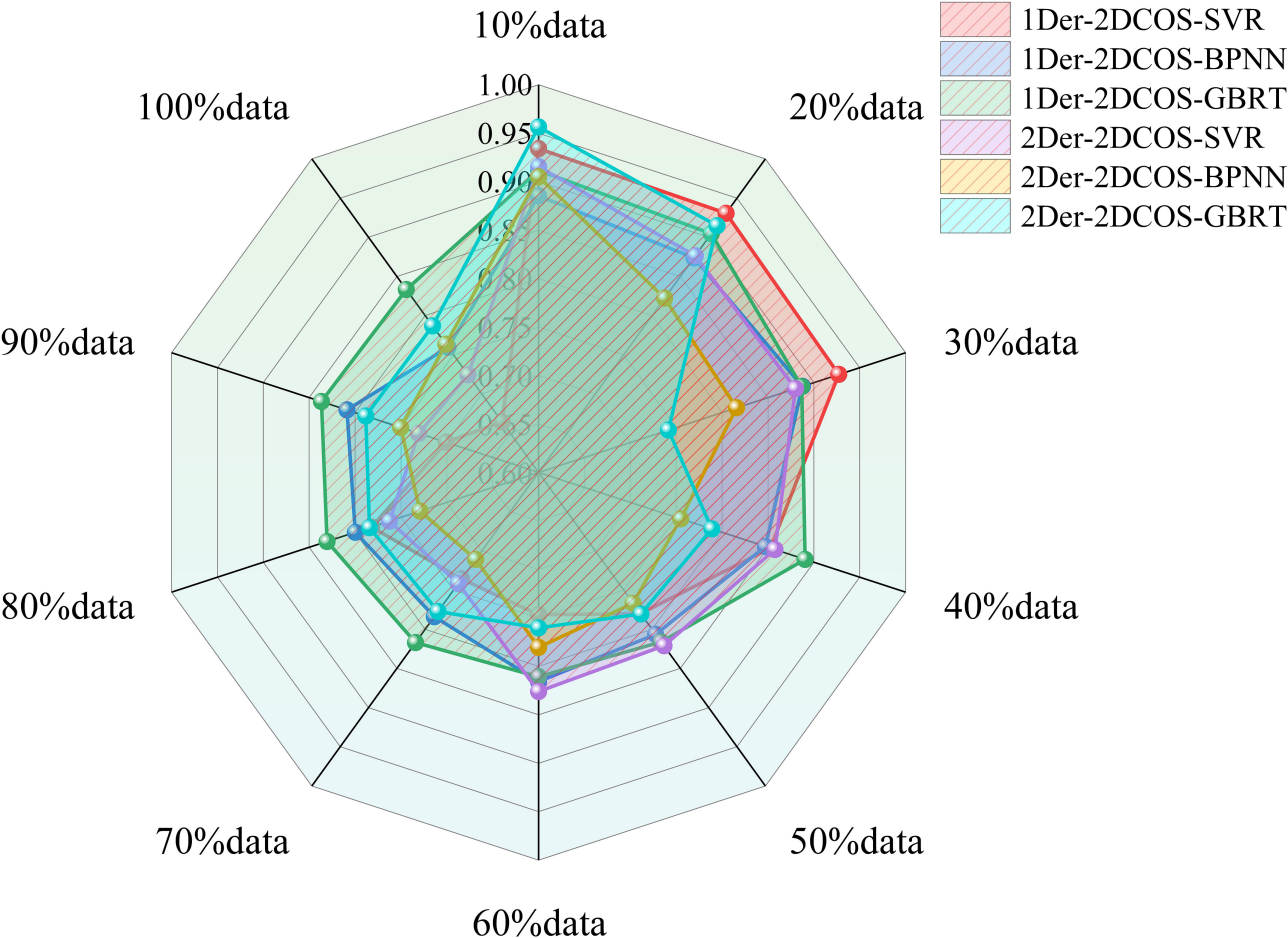
The prediction performance under the same sensitive frequency band.

We compared three different types of regression methods—Support Vector Regression (SVR), Back-Propagation Neural Network (BPNN), and Gradient Boosting Regression Tree (GBRT)—under varying data sizes. While the SVR and BPNN models perform well on small datasets, their predictive accuracy significantly decreases with larger datasets (as shown in **Figure 4**). When the data volume is less than 100, the results of 1Der-2DCOS-SVR are higher and the model is more stable. However, when the amount of data is large, the prediction effect is average. The 1Der-2DCOS-GBRT model is the most stable among these models. No matter how the size of the dataset changes, its prediction accuracy remains consistent, which can achieve more robust and accurate predictions. This result also solves the problem of using small data to predict.

### 3.2 Empirical model prediction of SPU

#### 3.2.1 Prediction of SPU based on overall plant characteristics

Predicting the SPU before the season helps to comprehensively understand the nutrient requirements and growth potential of crops, and can adjust the fertilizer application rate reasonably, thereby improving the efficiency of phosphorus fertilizer use and reducing carbon emissions in agricultural production. At the same time, pre-season forecasting can also help agricultural production respond quickly to uncertain climate conditions, improve crop stress resistance and stability, and promote sustainable agricultural development.

This study directly predicts SPU using spectral data from the jointing stage, filling stage, and milk stage. As shown in **Table 2**, the best period for pre-season prediction is the grouting period.

**Table 2:** SPU prediction results at different growth stages.

		R^2^_test_	RMSE_test_	MAE_test_	MAPE_test_
1Der-2DCOS-SVR	jointing	0.7757	2.3515	9.9863	0.2425
	**filling**	**0.8327**	**1.5253**	**4.0063**	**0.1267**
	milk	0.7883	2.1993	9.1119	0.2769
1Der-2DCOS-GBRT	jointing	0.7258	2.6399	2.0155	0.0634
	**filling**	**0.7351**	**4.9514**	**3.2085**	**0.0792**
	milk	0.6668	3.4575	2.9863	0.0963
1Der-2DCOS-BPNN	jointing	0.7284	5.0201	4.9882	0.1254
	**filling**	**0.7893**	**6.1996**	**10.0962**	**0.3105**
	milk	0.7254	3.9593	12.1049	0.3333

Pre-seasonal prediction of SPU is a key technology in precision agriculture management. It can not only improve the sustainability of agricultural production, but also provide important scientific basis for agricultural decision-making. It has profound significance for the intelligent management and resource optimization of modern agriculture.

#### 3.2.2 Prediction of SPU based on plant parts

Directly predicting the P uptake of the entire plant may overlook the differences between these parts, resulting in inaccurate predictions. By predicting the P uptake of different parts of maize plants, the model can flexibly adjust the parameters of each part, avoiding simplifying the entire maize’s P uptake process into a unified parameter. This type of model is easier to explain and optimize, and also helps to reveal the specific laws and influencing factors of P uptake between different parts, thereby more accurately integrating the effects of different factors on P uptake in various parts and improving prediction accuracy.

We selected the relatively important parts of corn plants - leaves, stems, and grains. However, traditional empirical models are difficult to distinguish the biomass of leaves and stems in canopy spectra. Therefore, we use the P concentration of mature leaves to represent the P concentration in the aboveground part of the plant, and multiply it with AGB except for grains to calculate the P uptake.

**Figure 5:**
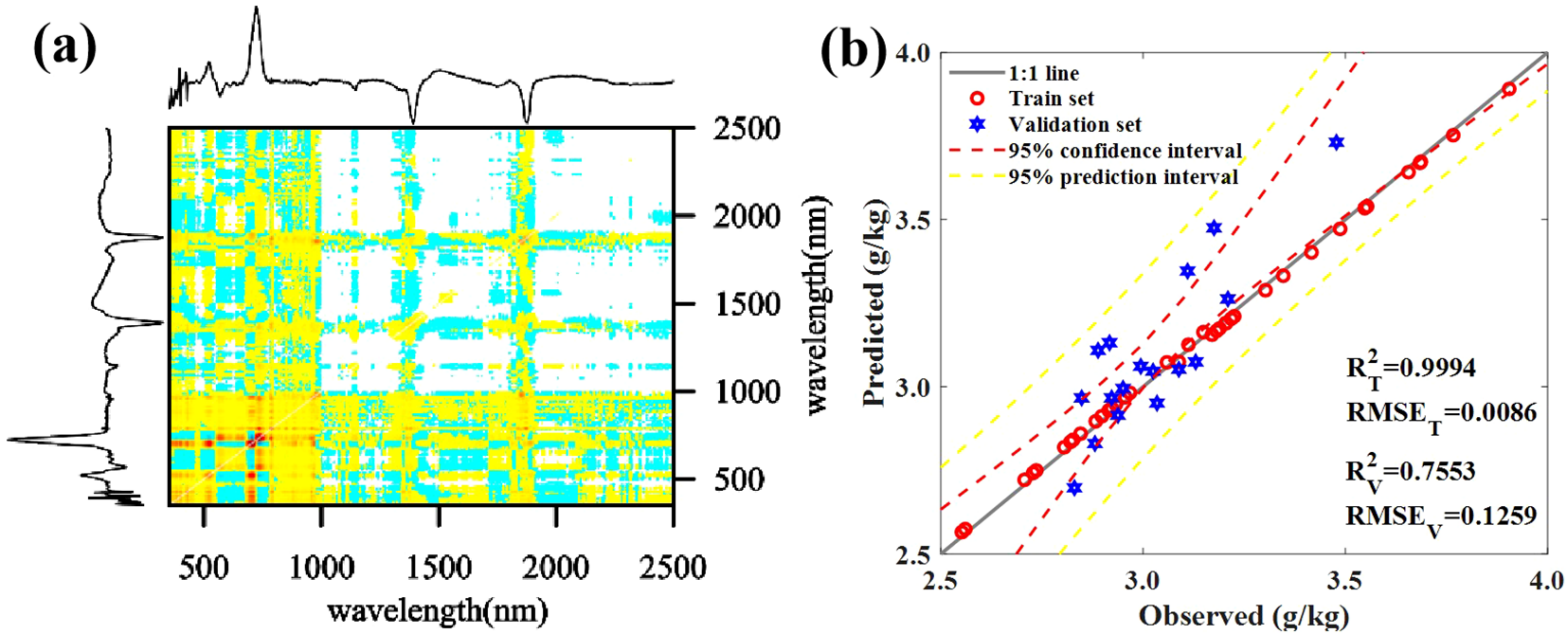
**(A)** Two-dimensional synchronous spectrum and **(B)** prediction results.

We applied the 1Der-2DCOS algorithm to the canopy spectrum to search for bands sensitive to the P concentration of mature leaves changes. Subsequently, we divided the dataset into training and validation sets in a 7:3 ratio to predict the P concentration in maize canopy leaves (n = 56). **Figure 5A** shows the 1Der-2DCOS analysis results for the canopy spectrum. **Figure 5B** shows the prediction results of the model, with a prediction accuracy reached 0.7553.

**Figure 6:**
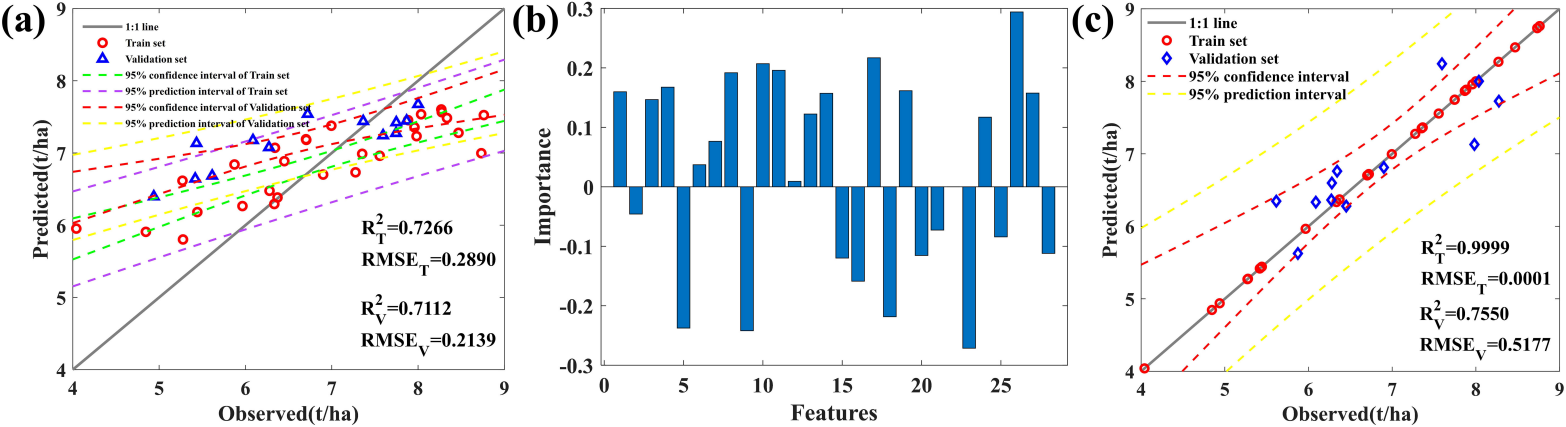
**(A)** Random Forest model. **(B)** The contribution of AGB sensitive bands. Features1-14: Filling Stage; Features15-28: Milk Stage. **(C)** AGB prediction model based on filling-milk-2T2DCOS.

At different growth stages, we studied and analyzed the correlation between different bands (see **Appendix S3.2.2** for specific bands) and AGB. **Supplementary Figure S1** shows that compared to the jointing stage, the correlation between maize AGB and spectral data is higher during the filling and maturity stages, as the jointing stage is in the early stages of growth and there is greater uncertainty in monitoring maize biomass. Therefore, in this study, the 2T2DCOS method was used to couple canopy spectral data during the filling and milk stages, namely filling-milk-2T2DCOS, where we selected over half of the samples (>20) as sensitive bands, i.e., 399, 400, 425, 553, 753, 1127, 1173, 1677, 1723, 2097, 2297, 2425, 2450, and 2451 nm. We established a random forest (RF) model (**Figure 6A**) and obtained the importance ranking of variables in RF (**Figure 6B**).

To avoid high correlation between the same frequency band at different growth stages, which may affect the model’s accuracy, we selected the growth period with high contribution from each band, i.e., F399, F753, F1173, F1677, F2097, F2297, F2451, M400, M425, M553, M1127, M1723, M2425, and M2450 (F: filling stage; M: milk stage). We constructed a dual-band index based on these 14 bands and ranked all the obtained variables’ correlations. We then selected the top 10 positively correlated bands and the top 10 negatively correlated bands, totaling 20 bands. Using an RF model to predict the AGB data (**Supplementary Figure S2**), we found an overestimation phenomenon in the simulation process of low biomass samples (AGB< 7 t/ha). Using the extreme learning machine (ELM) algorithm (see **Appendix S2.4.3**) to establish an AGB prediction model, we found that DSI has the best prediction effect (**Figure 6C**).

**Figure 7:**
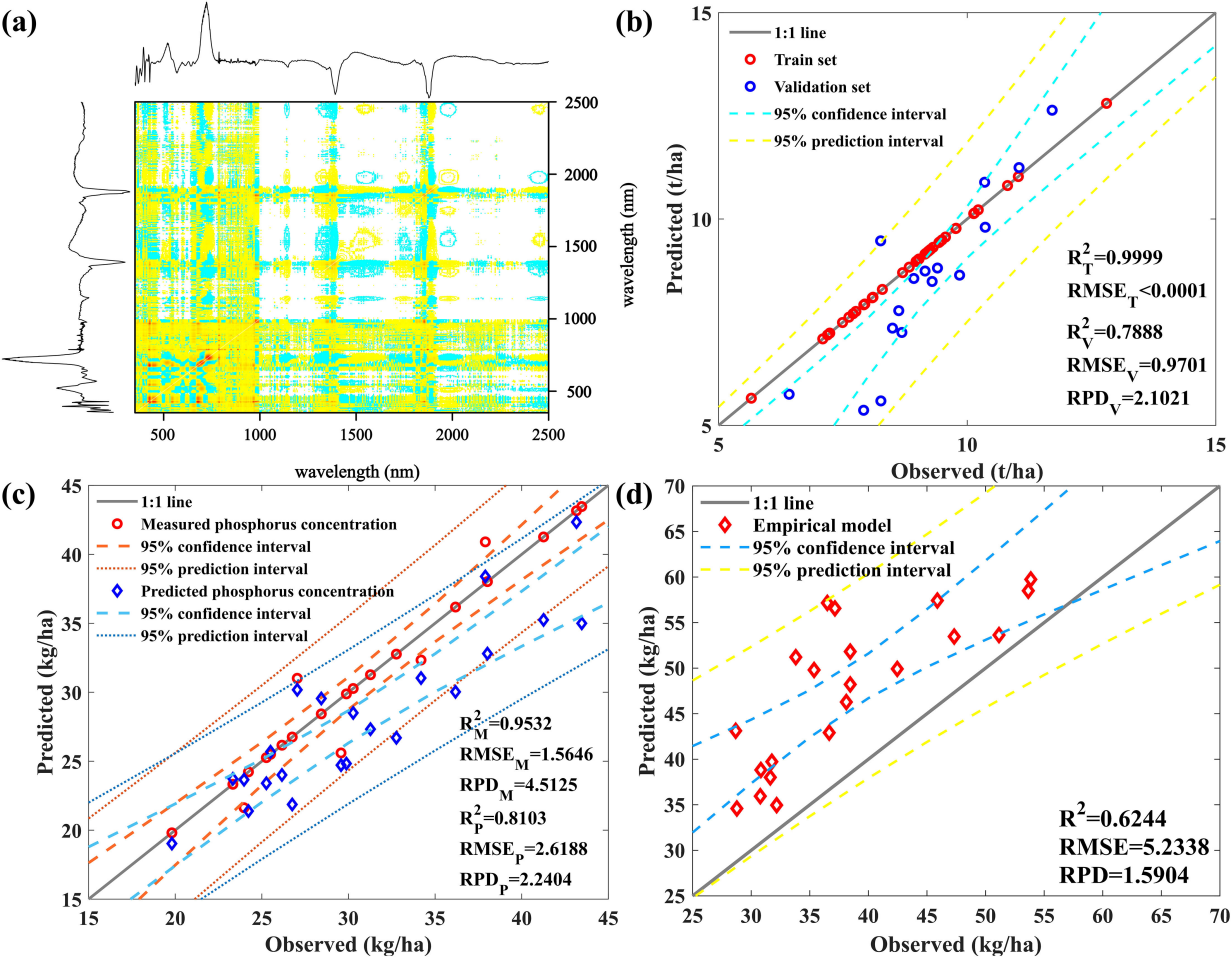
**(A)** The synchronous spectrum of the first-order differential spectrum; **(B)** The corresponding predicted grain biomass results; **(C)** Model for predicting P uptake by grains; **(D)** The prediction model results of SPU based on measured data.

During the filling stage, P stored in the nutrient organs is gradually reactivated into the developing grains to support their growth: 50%–85% of leaf P and 15%–50% of stem P accumulated before silking stage production are re-fixed into the grains (Zhang et al., 2022). Therefore, this study suggests that the P concentration in mature corn leaves is equal to that in harvested grains, and that the P concentration in mature corn leaves can be used instead of that in grains (Woli et al., 2018) (**Supplementary Figure S3**). Meanwhile, using the milk stage spectrum to predict grain biomass, as shown in **Figure 7A**, with the 1Der-2DCOS spectrum. We established a prediction model for grain biomass, as shown in **Figure 7B**, with great prediction performance (*R* ^2^ = 0.7888, RPD = 2.1021). Calculates grain P uptake by multiplying the two, as shown in **Figure 7C**, with *R* ^2^ = 0.8103 and RPD = 2.2404. The prediction effect is excellent, thus achieving nondestructive and high-precision prediction of grain P uptake based on remote sensing data.

From the perspective of reducing model computational complexity and saving computational time, we obtained two models for predicting SPU in maize by predicting LPC based on measured data from the jointing and filling stages, predicting AGB of maize except for grains based on measured data from the filling and milk stages, and combining them with the maize grain P uptake prediction method. **Figure 7D** shows that although the computational and time costs were reduced, the best-performing model only achieved an accuracy of 0.6244 at the cost of sacrificing the model’s accuracy, and the predicted values were generally overestimated.

### 3.3 Hybrid model prediction of SPU

#### 3.3.1 Parameter calibration

To improve the accuracy of the leaf biomass inversion model, we used the EFAST method for sensitivity analysis of the PROSAIL model parameters (**Appendix S3.3.1**) and calibrated the PROSAIL model parameters using the mixing sine and cosine algorithm with the Lévy flying chaotic sparrow algorithm, thus providing accurate model input parameters for maize planting areas under different treatments within the research area and applying them to leaf biomass inversion.

We set the sensitivity threshold to 0.1 and considered parameters above this threshold as high-sensitivity parameters. The results of the sensitivity analysis indicate that during the filling stage, leaf area index (LAI), leaf chlorophyll content (C_ab_), and average leaf angle (ALA) considerably affect visible light. LAI and ALA considerably impact near-infrared spectra. LAI ALA, and leaf water content (C_w_) considerably impact shortwave infrared spectra. Compared with the filling period, the sensitivity analysis of the green light band and shortwave infrared 1 during the milk stage has changed. During the milk stage, the sensitivity of the total carotenoid content to the green light band is relatively high, and the hot-spot parameter (hs) also has a certain impact on the shortwave infrared spectrum. Ultimately, input parameters with strong global sensitivity include LAI, C_ab_, C_w_, total carotenoid content (Car), hs, and ALA. Although dry matter content (C_m_) is not a sensitive variable, in this study, the PROSAIL model was mainly used for simulating leaf biomass; therefore, this variable was also included as a sensitive variable.

Based on this preliminary analysis, we have decided to only change the most sensitive parameters, which have a wide range of amplitudes and sensitivities, while keeping other parameters unchanged, which is the standard value in **Supplementary Table S2**. This decision seems reasonable considering the required computing power and a single crop in the same region, which is the focus of this study.

**Supplementary Figure S6** shows poor simulation results at 400–615 nm and 765–990 nm. Therefore, we selected simulation datasets of 615–765 nm and 990–2500 nm for subsequent modeling.

#### 3.3.2 Leaf biomass prediction

Using parameter calibration results, we established a quantitative relationship between reflectance and LAI, C_m_, using the PROSAIL model to develop a remote sensing inversion model for maize leaf biomass. Based on a simulation dataset of 59,400, we established a plant leaf biomass prediction model for the top 30 bands of RF selection variable importance ranking. This model performs very well on the prediction dataset, with fitting accuracy and RMSE of 0.88 and 0.21 t/ha, respectively. However, when validating the model with field measurement data, the effect was not satisfactory (as shown in **Supplementary Figure S7**, *R* ^2^ = 0.15, RMSE = 0.19) because of the “same effect with different parameters” phenomenon and parameter combinations that do not match the actual situation used for simulation in the model, which may lead to substantial deviations between the inversion results and the measured values. Therefore, the purposeful selection of samples to establish models is crucial.

**Figure 8:**
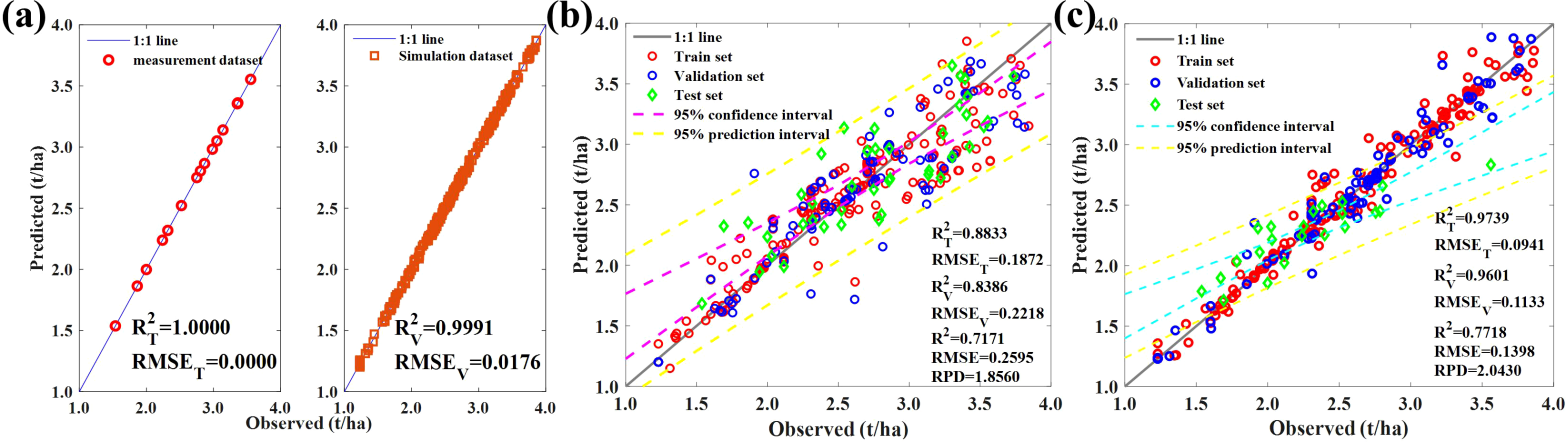
**(A)** The effectiveness of NRBO-AL’s sampling strategy. The leaf biomass prediction model in **(B)** Experiment 1 + 2 and **(C)** Experiment 1 is based on NRBO-AL correction.

The physical method process is complex, with too many parameters, making the inversion of the model ill-posed. Ill-posedness refers to the possibility that different parameter combinations may result in the same canopy reflectance, leading to the best-matched simulated reflectance not necessarily resulting in the best canopy parameters. Therefore, we attempted to use active learning strategies to filter the simulated databases. Based on the NRBO optimization simulation dataset, we obtained 200,000 pieces of data. We then established a linear regression model using 35% of the measured data and selected 464 data points with better performance from the simulated dataset according to the sampling strategy of NRBO-AL as the updated training data (**Figure 8A**).

**Figure 8B** shows the results of using the training dataset to establish an ELM model for predicting the measured leaf biomass data in the field. Active learning can effectively improve the effectiveness of PROSAIL models and enhance the effectiveness of simulated training sets, with *R* ^2^ = 0.72 and RPD = 1.86, indicating that high-precision estimation of maize leaf biomass can be achieved through active learning. Using 464 training data points, we re-predicted the data from experiment 1, and the results are shown in **Figure 8C**. The prediction effect was good: *R* ^2^ = 0.77, RMSE = 0.14, and RPD = 2.04.

#### 3.3.3 SPU prediction

**Figure 9:**
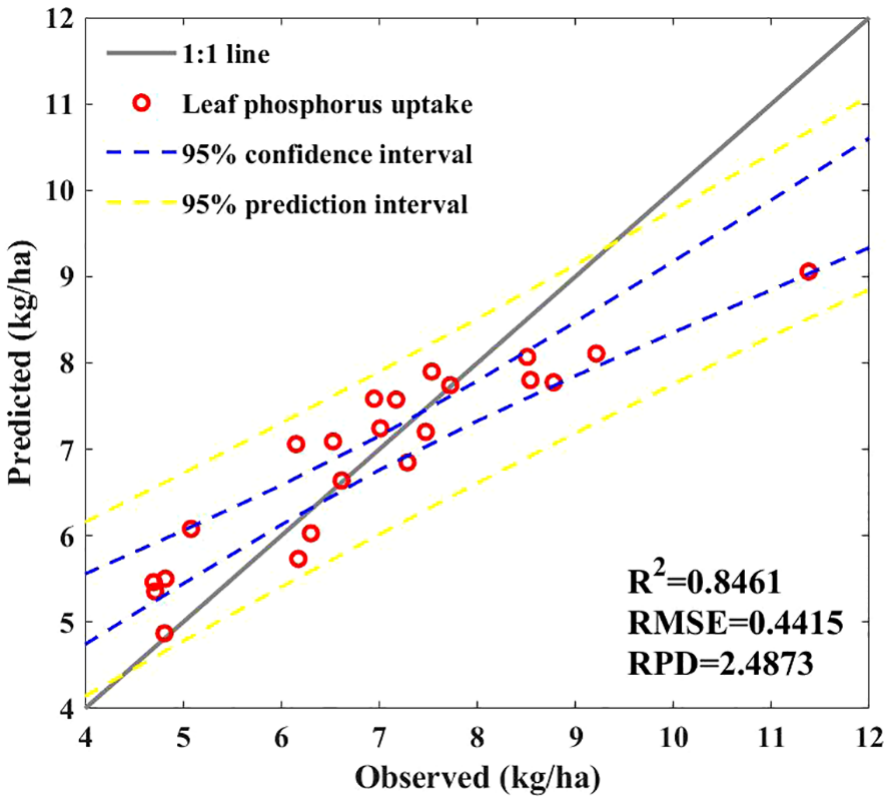
Predictive models for P uptake by leaves.

We estimated the leaf P uptake of maize leaves by multiplying the canopy P concentration of maize plants by the leaf biomass predicted based on NRBO-AL. **Figure 9** shows that the model’s prediction accuracy can reach 0.85, with an RPD > 2, indicating good prediction performance. However, the predicted interval shows that when leaf P uptake is >9 kg/ha, the predicted value is slightly lower than the true value.

**Figure 10:**
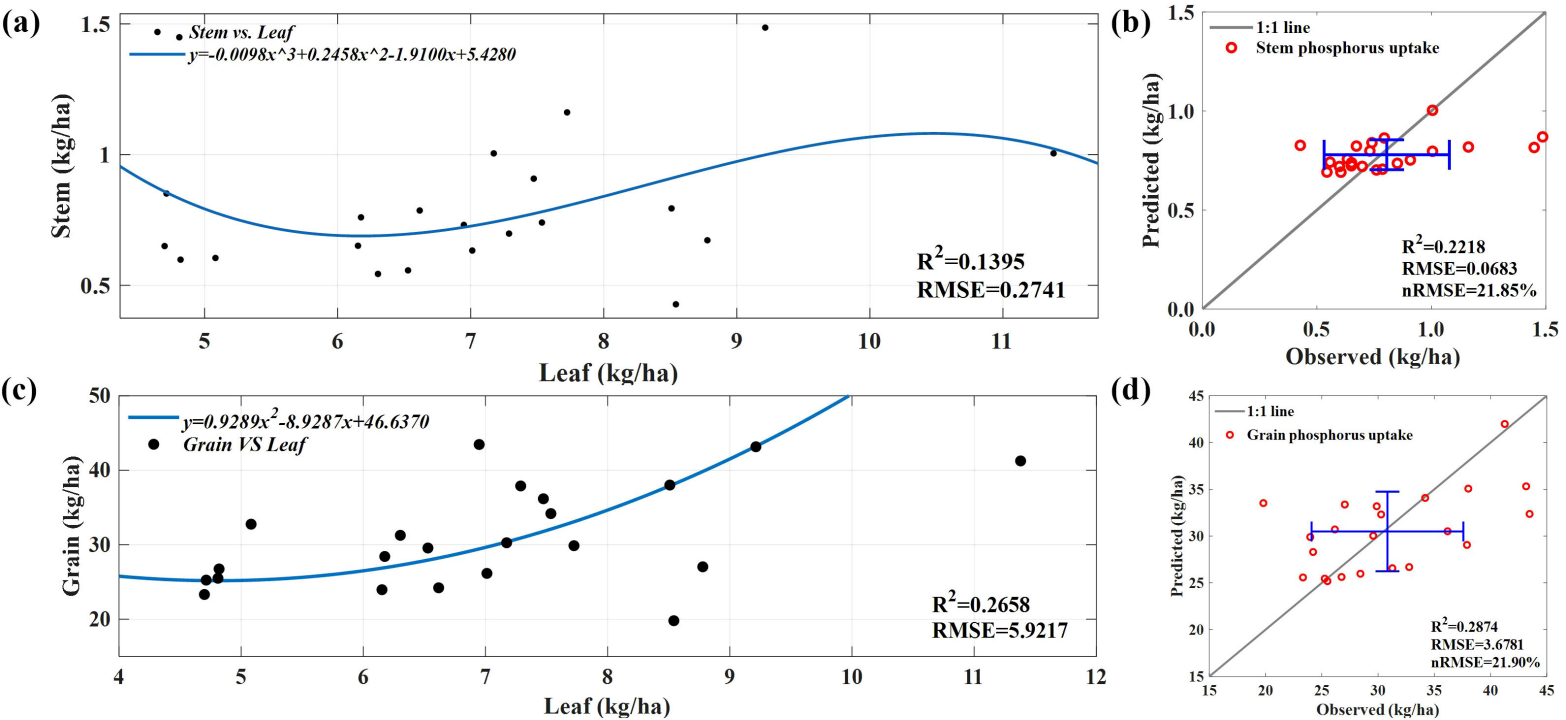
**(A)** A fitting model between the measured P uptake by leaves and the measured P uptake by stems. **(B)** Predict the P uptake by the stem. **(C)** A fitting model between the measured P uptake by leaves and the measured P uptake by grains. **(D)** Predict the P uptake by grains.

In general, because straw mainly comprises non-photosynthetic tissues such as stems, leaf sheaths, and tassels, the stem P uptake of straw is relatively low, and its metabolic activity is relatively low. In addition, because of the difficulty in monitoring the P concentration and biomass of corn stems through spectroscopy, we attempted to use polynomial regression to fit the relationship between the measured leaf P uptake and the measured stem P uptake (**Figure 10A**) and applied the model to the predicted leaf P uptake data. **Figure 10B** shows that the predictive performance of polynomial regression is average, and the predicted values are relatively concentrated, mainly between 0.70 and 0.85 kg/ha. However, regarding absolute RMSE (Wocher et al., 2022), RMSE of stem P uptake decreased by 75% compared with the training results.

The relationship between leaf and stem P uptake is complex and diverse (Poorter et al., 2012). Because of the synergistic effect between leaves and stems in absorbing and utilizing P elements, we attempted to indirectly predict the amount of P absorbed by stems using remote sensing methods and solved the limitations of remote sensing data usage. However, the prediction effect is poor, and the predicted values are relatively concentrated because of the way leaves and stems absorb and utilize P during plant growth and development. Predicting grain P uptake is based on polynomial regression to fit the relationship between measured leaf P uptake and grain P uptake (**Figure 10C**) and is applied to the predicted leaf P uptake dataset. **Figure 10D** shows that in terms of absolute RMSE; RMSE of the grain P uptake prediction model decreased by 38% compared with the training results, indicating poor performance.

**Figure 11:**
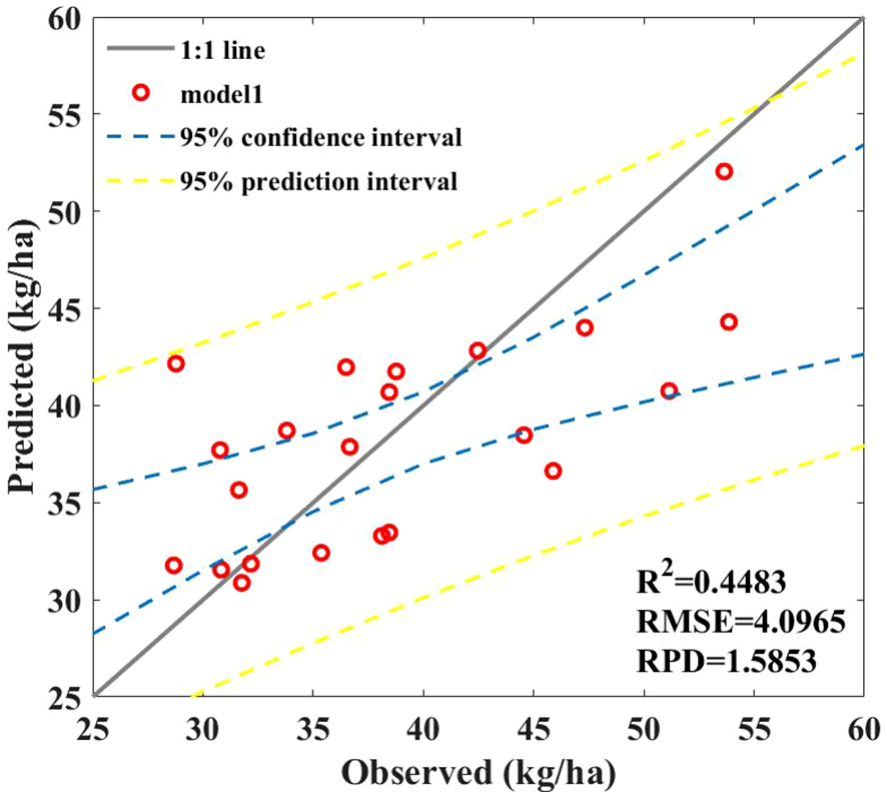
Results of SPU prediction model based on hybrid model.

The final SPU prediction model is obtained by combining the P uptake of leaves based on a hybrid model and the P uptake of stems and grains obtained from this model, also referred to as the hybrid model. Although the prediction of P uptake by leaves is effective, polynomials cannot fully demonstrate the complex dynamic relationship between P uptake by maize leaves, stems, and grains. Therefore, the final effect is poor, as shown in **Figure 11**, with the *R* ^2^ of only 0.45.

### 3.4 Combined model prediction of SPU

**Figure 12:**
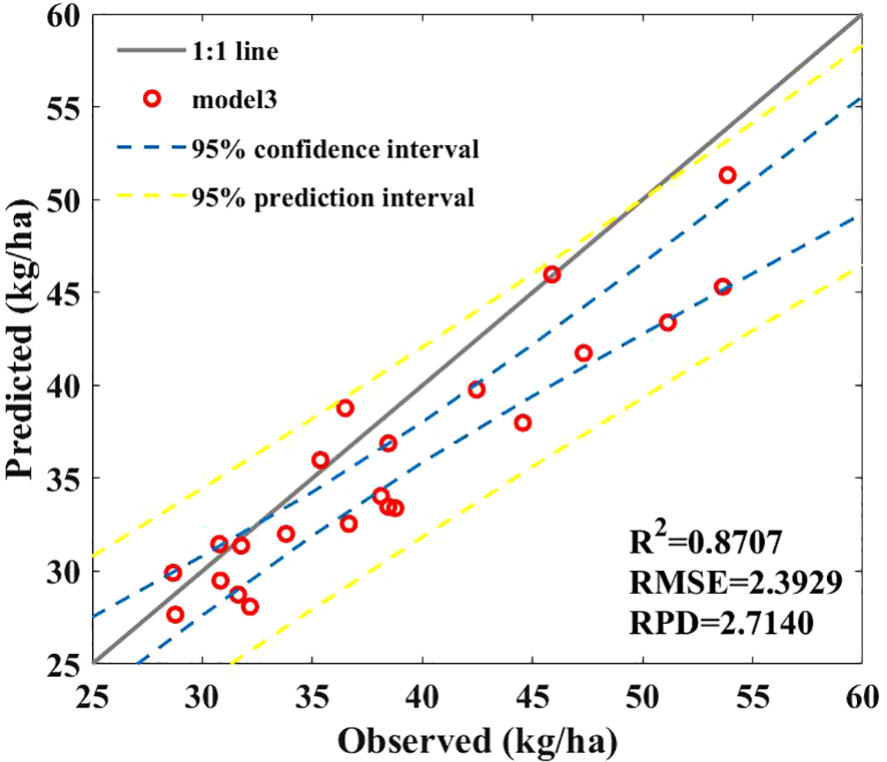
Results of SPU prediction model based on combined model.

We established a combined model to predict corn SPU based on the above research results. Combining the advantages of empirical and hybrid models in SPU prediction, the combined model consists of a leaf P uptake prediction model, a stem P uptake prediction model in the hybrid model, and a grain P uptake model in the empirical model. **Figure 12** shows that the combination model achieved the best performance. Although the introduction of the NRBO-AL sampling strategy greatly increases the computational complexity and time cost of the model, the accuracy of the prediction model has also been considerably improved (*R* ^2^ = 0.8707, RPD = 2.7140), a 24.63% increase.

Based on the combined prediction model, it is possible to achieve nondestructive prediction of SPU in maize plants based on hyperspectral data, which will provide important guidance for timely P nutrition management decisions.

## 4 Discussion

### 4.1 The potential of 2DCOS and 2T2DCOS

Compared with visible and near-infrared spectra, 2DCOS and 2T2DCOS are advanced spectroscopic analysis techniques with wider applications in infrared, fluorescence, and Raman spectra (Noda, 2017; Li et al., 2021a). Nevertheless, researchers such as Zhang et al. (2021a) have proposed using shape- and amplitude-enhanced 2DCOS combined with transfer learning to estimate chlorophyll content in winter wheat. Zhang et al. (2024) used a 2DCOS analysis method based on first-order differential enhancement to highlight chlorophyll information in the spectrum. These results indicate that 2DCOS has enormous potential in visible/near-infrared remote sensing.

**Figure 13:**
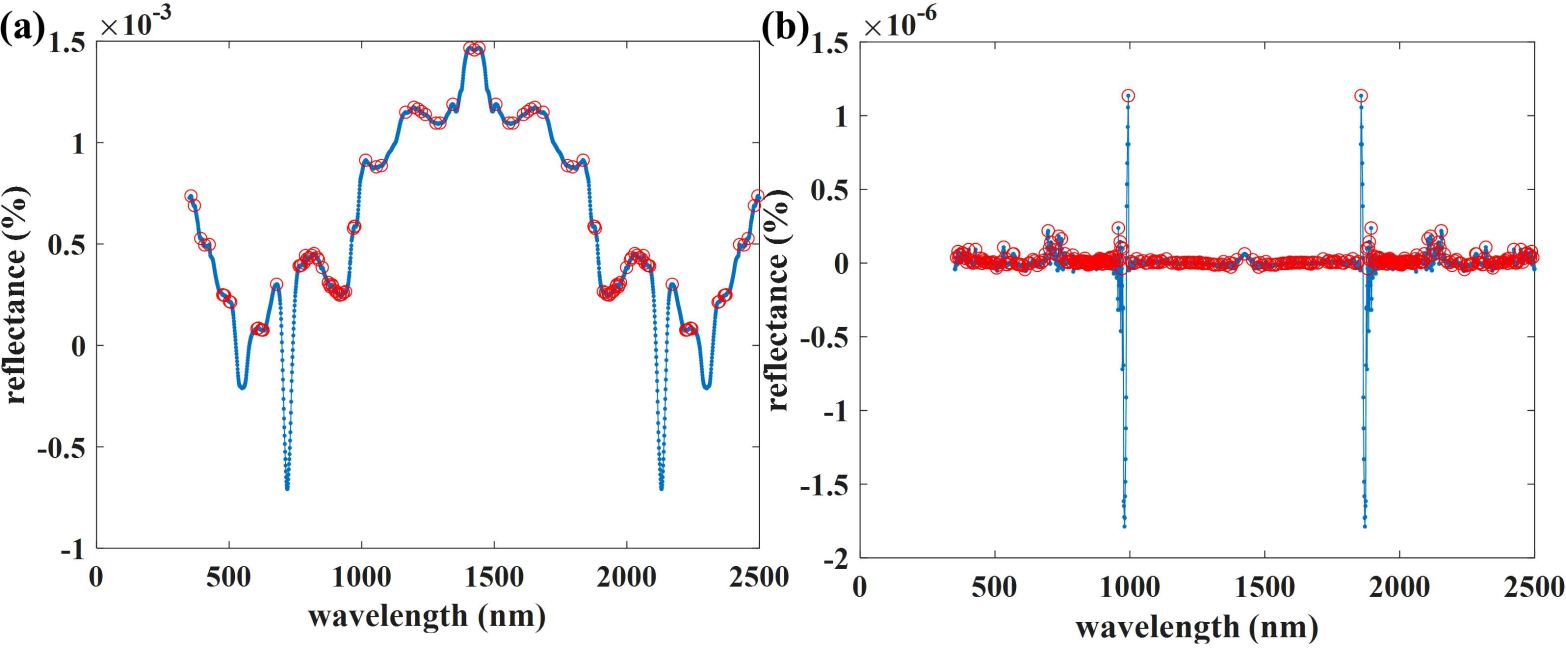
Autocorrelation peak extracted from the **(A)** 2DCOS synchronous spectrum based on the original canopy spectrum and **(B)** 1Der-2DCOS synchronous spectrum.

Our study found that compared to the Raw-2DCOS, baseline-corrected 2DCOS can effectively improve the accuracy of the prediction model (15–17%) (**Supplementary Figure S8**). This improvement is due to the narrowing of the absorption peak in the first-order derivative spectrum and the sharpening of the peak shape after derivative conversion, this method extends traditional spectra in two dimensions and improves the screening ability of sensitive bands based on first-order differential spectra, thereby obtaining more effective sample information (Dong et al., 2021). Although the peak of Raw-2DCOS is clear and the feature information is relatively intuitive, based on the full band spectrum, 1Der-2DCOS displays subtler feature information, including the number of self-peaks and cross-peaks, peak intensity, and their correlation (**Figure 13**). These are helpful for the attribution analysis and sample identification analysis of different peaks.

Although biomass prediction models vary under the influence of different external factors (**Figures 6**, **7**), small sample datasets can still provide predictions with sufficient accuracy in this variability. To better characterize the relationship between spectra at different growth stages and AGB, we proposed a 2DCOS analysis method that couples filling and milk stages. **Supplementary Figure S9** shows the autocorrelation peak extracted from the filling-milk-2T2DCOS synchronous spectrum, where the peak position is relatively stable. Therefore, this method performs well on small datasets, with a high repetition rate of the selected sensitive bands and good universality.

Our experiment is based only on ground-scale data from the fields used to construct the models. In the next step, we will use the small dataset model of this study to accurately monitor and predict satellite remote sensing data, reduce the cost of on-site sampling, provide a scientific basis for management decision-making in agriculture, the environment, and other fields, and promote the application and development of remote sensing technology in P uptake in aboveground parts of farmland.

### 4.2 Limitations in predicting P uptake by stems

Research has shown a synergistic effect between leaves and stems in absorbing and using P elements (Wu et al., 2015). For example, increasing the P uptake capacity of leaves may promote the transportation and use of P in stems, thereby improving the overall P utilization efficiency of plants. In contrast, the storage and transportation of P in stems may also affect the uptake and utilization of P by leaves, and there is an interactive relationship between the two. Therefore, we conducted a correlation analysis between the two to predict the P uptake of corn stalks without any damage.

However, the relationship between P uptake by maize leaves and stems is not constant but varies with growth stage and environmental conditions (Zhang et al., 2022).

First, there are substantial differences in the function and tissue structure of leaves and stems during plants’ growth and development stages, which leads to different ways in which they absorb and utilize P (Adam et al., 2018). In general, leaves are the main organs for photosynthesis (Wu et al., 2019); therefore, a high demand for P is required for photosynthesis. Relatively, the stem mainly plays a role in supporting and transporting nutrients (Wang and Ning, 2019), and its demand for P is relatively low. Therefore, during the growth process of plants, leaves usually absorb and utilize P more actively than stems. Second, plant growth stage and external environmental factors influence the relationship between leaf P uptake and stem P uptake (Marschner et al., 1996) and external environmental factors (Bi et al., 2020; Mao et al., 2021). In the early plant growth stages, the growth rate of leaves is relatively fast; therefore, their demand for P is also relatively high (Kerkhoff et al., 2006). As the plant grows, the growth rate of the stem gradually accelerates, and its demand for P will also gradually increase. Therefore, to understand the relationship between P uptake by corn leaves and straw, we must consider other factors, such as plant growth stage and environmental factors.

We only investigated the P uptake by leaves and stems during one stage of growth and development without considering the influence of external factors; thus, we could not fully interpret the complex and diverse relationship between leaf P uptake and stem P uptake. Although the stem P uptake prediction results in this study are generally low, the impact on the overall SPU is relatively small. If this relationship can be further studied in the future, it will help to better understand the demand and utilization patterns of P in plants, provide a scientific basis and technical support for agricultural production, and have important significance in plant growth and development and P nutrition management.

### 4.3 Impact of interannual planting on SPU

P plays a crucial role in various physiological processes in plants (Lambers, 2022; Khan et al., 2023), including energy transfer and nucleic acid synthesis. However, its availability in soil is often low (Gao and DeLuca, 2018; Cao et al., 2021), which limits the efficiency of plant uptake. As a result, environmental factors (Song et al., 2020) and cultivation practices (Zhang et al., 2016) significantly influence P uptake. Additionally, the impact of interannual planting, as a relatively long-term cultivation factor, can also affect P uptake and utilization.

With interannual planting, the availability of P in the soil may be affected by processes such as annual fertilization (He et al., 2018), adaptability of plant roots (Liu, 2021) and soil P conversion (Hussain et al., 2023; He et al., 2018). Long-term application of chemical phosphorus fertilizers can lead to the accumulation of phosphorus in the soil, particularly when soil phosphorus levels reach saturation. This may reduce the plant’s ability to absorb phosphorus. Additionally, the continuous cultivation of certain crops, especially those with high phosphorus requirements, can result in the gradual depletion of soil phosphorus over the years, potentially impacting phosphorus uptake in subsequent seasons.

Therefore, proper cultivation practices such as planting density (Luo et al., 2023; Yan et al., 2024), irrigation methods (Li et al., 2021b; Wang et al., 2021), and fertilization management can improve P availability in the soil and enhance plant P uptake. When planting density is high, root space is limited, which can increase competition among roots and reduce their ability to absorb P. Proper density management can mitigate this issue and improve P use efficiency in the soil. Irrigation has a significant impact on P uptake (Wang et al., 2021); excessive irrigation can lead to P leaching or loss, particularly when soil P levels are high. On the other hand, insufficient irrigation can cause soil water stress, restricting the plant’s ability to absorb P. Thus, effective irrigation management not only improves water use efficiency but also optimizes P uptake. The application method and timing of P fertilizer directly affect plant P uptake. Therefore, it is necessary to forecast P uptake in the above-ground plant parts before the season and implement integrated measures based on local soil conditions and crop types (Lyu et al., 2016) to ensure the efficient use of P fertilizers.

### 4.4 Performance of the empirical and hybrid models

In this study, we used a combined prediction model to improve the accuracy of inverting maize SPU from field spectral data and compared its performance with that of empirical methods and a hybrid model. Overall, the combination model has the highest accuracy and is superior to the empirical models (**Figure 7**) and hybrid models (**Figure 11**). This may be because, in the empirical model, we established a relationship between the sum of the biomass of organs (such as leaves, stems, and axes) and the spectra. Because of crop canopy obstruction, the response of spectral reflectance to AGB may only be concentrated on the biomass of canopy leaves and cannot fully extract effective information from other organs, resulting in slightly poor prediction performance.

The hybrid model based on NRBO-AL constraints also performs better than the general hybrid models (**Supplementary Figures S7**, **S8**). When there are many unknown variables in the search space of the PROSAIL model, RTM inversion makes it challenging to accurately infer the biophysical variables of corn (Liang et al., 2015; Danner et al., 2021). Although prior knowledge under the constraint of actual measurement range increases the feasibility of hybrid methods for simulating plant growth states, it cannot replace real datasets. This may also be why despite limiting the search space for highly sensitive parameters in the PROSAIL model, the results are still not as accurate as those of the hybrid model based on active learning correction (**Supplementary Figure S7**).

To address this issue, we combined active learning with hybrid methods to improve the estimation of leaf biomass through canopy reflectance. Our results indicate that compared with random selection, the NRBO-AL sampling strategy can select more representative samples in each iteration and exhibit better distribution in the sample space, with higher diversity and representativeness (**Supplementary Figure S10**). As the number of simulated data increases, this difference may become more pronounced, and the larger the randomly selected sample size, the more errors and uncertainties it may bring (Verrelst et al., 2016).

Féret et al. (2021) reported that the scale, reflectance anisotropy, and canopy structure from leaves to canopy can affect inversion accuracy. However, we improved the prediction accuracy of SPU and reduced the uncertainty level of the simulated dataset using a hybrid model based on active learning correction. This indicates that the model’s predictive performance can achieve consistent results under different environmental conditions.

Therefore, using high-quality and informative samples is crucial instead of numerous training datasets (Verrelst et al., 2016), as this will reduce costs and computational requirements and improve model performance. This requires selecting sufficiently representative data (Hauser et al., 2021) to fully cover the possible range of these variables, i.e., an active learning method based on the NRBO-AL sampling strategy and selecting high-quality training sets from a large number of simulated datasets. Our work will drive further research on biomass prediction models using the PROSAIL model.

### 4.5 Implications for future work

Although *in situ* samples are still required for predicting P uptake in combined models, our results show that active learning and two-dimensional spectral analysis require fewer samples to achieve excellent model performance compared with traditional empirical prediction models. Therefore, this study can reduce sampling costs and establish an estimation model by reducing the number of *in situ* samples. In practical situations, it is sufficient to select representative samples at critical reproductive stages; therefore, there is no need to accumulate many on-site samples for model training.

In addition, our model performs very well in monitoring P uptake in maize using remote sensing data from scientific experimental area-scale experiments. If new samples from different experiments can be combined, active learning can continuously improve the model. This continuous updating mechanism is highly robust under different plant species and environmental conditions. Therefore, in the future, we will collect ground measurement samples and remote sensing satellite images from different regions, experimental treatments, and future crops, calibrate the model to local crops, and provide spatial distribution predictions of P uptake over a large area. However, an important source of uncertainty in applying this method to large-scale farmland is the relatively large spatial variability of P observed in small-scale crops on the planting site.

To train and validate models using satellite remote sensing data in this situation, in our future work, we will (i) determine pixel sizes smaller than the spatial variability, (ii) collect accurate samples and image localization, and (iii) select sampling areas with minimal spatial variability. We also suggest that further research should include the evaluation of P uptake prediction models for large-scale farmland to evaluate the accuracy of these models in practical applications.

## 5 Conclusions

In optimizing fertilization management and protecting the environment, monitoring the surplus of soil P is crucial and largely depends on the timely monitoring and application of crop P. In this study, we developed three methods to monitor SPU of maize using field spectroscopy, including empirical, hybrid, and combined methods. The main findings of this study are as follows.

1. Based on two-dimensional correlation spectra, we can achieve stable screening of sensitive bands in small datasets, in which the sample size of the dataset does not limit the number of selected bands.
2. The quality of the simulated datasets in the hybrid models considerably impacts the prediction performance. The accuracy of boundary constraint models based on actual situations is not as good as that based on NRBO-AL sampling constraints. This may be because the active learning strategy removes simulated data that differ considerably from the actual situation.
3. The filling stage is the optimal time to directly predict maize SPU using canopy reflectance spectra. If the SPU of maize is calculated in different parts, the P concentration of mature leaves in the canopy can be used to replace the P concentration in grains to predict the P uptake by grains.
4. The combination model of leaf biomass prediction model based on NRBO-AL sampling constraint, LPC and grain biomass prediction model based on 1Der-2DCOS, and polynomial prediction model of stem P uptake is a robust method for estimating maize SPU, as it helps to solve the key limitations of RTM and empirical methods while maintaining their key advantages.

Spaceborne hyperspectral sensors can provide a unique environmental process detector on a global scale. To ensure optimal use of such a rich dataset, we anticipate that the combination prediction model (described in this study) will play a crucial role. This study provides new ideas and evaluation tools for the large-scale analysis of aboveground P uptake in crops. Continuous monitoring of P removal from crop aboveground parts on a global scale will promote the optimization of fertilizer application and other environmental sustainability goals. Ultimately, continuous monitoring of P removal from crop aboveground parts can be achieved globally, promoting the development of agriculture toward efficiency, environmental protection, and sustainability.

## Data Availability

The raw data supporting the conclusions of this article will be made available by the authors, without undue reservation.
