# Supplementary material for: A combined model of shoot phosphorus uptake based on sparse data and active learning algorithm
Source: Front Plant Sci. 2025 Jan 22;15:1470719. doi: 10.3389/fpls.2024.1470719 (PMC11794547; doi:10.3389/fpls.2024.1470719)
Supplement: Supplementary file 1 [file DataSheet1.pdf]

*Supplementary Material*

# Realizing Sustainable Phosphate Fertilizer Utilization: A New Framework and Case Study for Predicting Shoot Phosphorus Uptake in Farmland

Tianli Wang, Yi Zhang, Haiyan Liu, Fei Li, Dayong Guo, Ning Cao*, Yubin Zhang*
*Correspondence: cao_ning@jlu.edu.cn, ybzhang@jlu.edu.cn

## S2. Materials and methods

## S2.2 Newton-Raphson-based optimizer

The Newton Raphson Based Optimizer (NRBO) (Sowmya et al., 2024) is a novel metaheuristic algorithm.

Step 1: Population initialization:

NRBO initiates the search for the optimal solution by generating an initial random population within the boundary of the candidate solution. Assuming there are $N_p$ populations, generate a random population within the boundary:

$$x_j^n = lb + rand \times (ub - lb), lb \le x_j \le ub \quad (1)$$

wherein, $n = 1,2,\cdots,N_p$; $j = 1,2,\cdots,dim$; $X_{ij}$: the position of the $j$-th dimension of the $n$-th population; rand: random numbers between (0,1).

Step 2: Newton-Raphson search rules (NRSR):

Update the position of the optimal solution based on NRSR to promote search trends and accelerate convergence.

$$x_n^{IT+1} = m \times (m \times X1_n^{IT} + (1-m) \times X2_n^{IT}) + (1-m) \times X3_n^{IT} \quad (2)$$

$$X1_n^{IT} = x_n^{IT} - NRSR + (a \times (X_b - X_n^{IT}) + b \times (X_{r_1}^{IT} - X_{r_2}^{IT})) \quad (3)$$

$$X2_n^{IT} = X_b - NRSR + (a \times (X_b - X_n^{IT}) + b \times (X_{r_1}^{IT} - X_{r_2}^{IT})) \quad (4)$$

$$X3_n^{IT} = X_n^{IT} - \delta \times (X2_n^{IT} - X1_n^{IT})), \delta \in [-1,1] \quad (5)$$

$$\delta = \left(1 - \left(\frac{2 \times IT}{MaxIT}\right)\right)^5 \quad (6)$$

wherein, IT: the current iteration; MaxIT: the maximum number of iterations; $m$: a random number between (0,1), a and b are random numbers between (0,1), $r_1$ and $r_2$ are different integers randomly selected from the population, and the values of $r_1$ and $r_2$ are not equal.

$$NRSR = randn \times \frac{(y_w - y_b) \times \Delta x}{2 \times (y_w + y_b - 2 \times x_n)}, \Delta x = rand(1, dim) \times |X_b - X_n^{IT}| \quad (7)$$

$$y_w = h \times \left(Mean\left(2x_n - randn \times \frac{(X_w - X_b) \times \Delta x}{2 \times (X_w + X_b - 2 \times x_n)}\right) + h \times \Delta x\right) \quad (8)$$

$$y_b = h \times \left(Mean\left(2x_n - randn \times \frac{(X_w - X_b) \times \Delta x}{2 \times (X_w + X_b - 2 \times x_n)}\right) - h \times \Delta x\right) \quad (9)$$

wherein, randn: a normally distributed random number with a mean of 0 and a variance of 1;

$X_b$: the best solution obtained so far; and $X_w$: the worst position; H: a random number between (0,1).

Step 3: Trap avoidance operator (TAO):

The inclusion of TAO is to avoid falling into local optima and improve the effectiveness of NRSR in handling practical problems.

$$\begin{cases} X_{TAO}^{IT} = X_n^{IT+1} + \varphi, if\ \mu_1 < 0.5 \\ X_{TAO}^{IT} = x_b + \varphi, Otherwise \end{cases} \quad (10)$$

$$X_n^{IT+1} = X_{TAO}^{IT} \quad (11)$$

$$\varphi = \theta_1 \times (\mu_1 \times x_b - \mu_2 \times X_n^{IT}) + \theta_2 \times \delta \times (\mu_1 \times Mean(X^{IT}) - \mu_2 \times X_n^{IT}) \quad (12)$$

wherein, $\theta_1$ and $\theta_2$are uniformly random numbers between (−1,1) and (−0.5,0.5), respectively, $\mu_1$ and $\mu_2$ is a random number, generated by formulas (13) and (14) respectively:

$$\mu_1 = 3 \times rand \times \beta + (1 - \beta) \quad (13)$$

$$\mu_2 = rand \times \beta + (1 - \beta) \quad (14)$$

$$\beta = \begin{cases} 0, \Delta \geq 0.5 \\ 1, Otherwise \end{cases} \quad (15)$$

Due to parameters $\mu_1$ and $\mu_2$ are the randomness of two choices makes the population more diverse and escapes from local optima, which helps to improve its diversity.

# S2.3.1 2T2DCOS

2T2DCOS, an advanced spectral analysis technique, is the construction of 2DCOS using a collection of two one-dimensional spectra, which is very different from the generalized 2DCOS. Compared with traditional differential and weighted spectrum subtraction techniques, the 2T2DCOS analysis technique can provide additional information (Noda, 2018). In this technique, only a pair of spectra is needed to obtain the relevant spectra, and no disturbance is required. In this study, we used the 2T2D correlation spectrum to couple the canopy spectra of the two growth stages, capturing rich and useful feature information and identifying spectral bands with synchronous changes.

The formula for constructing the synchronous 2T2D correlation spectra is as follows:

$$\emptyset(v_1, v_2) = \frac{1}{2}[A(v_1) \cdot A(v_2) + B(v_1) \cdot B(v_2)] \quad (16)$$

where *A* and *B* represent the canopy spectra at different growth stages.

# S2.3.2 2DCOS

LPC change is considered to be an external interference to the maize canopy spectral system, and the corresponding spectrum is defined by Noda (1993) as a dynamic spectrum. In 2DCOS, obtaining the intensity of 2DCOS as two independent spectral variables is shown in Eq. (1). We obtained synchronous and asynchronous 2DCOS using a fast Fourier transform of the dynamic spectrum, represented by real and imaginary components:

$$X(v_1, v_2) = \emptyset(v_1, v_2) - i\psi(v_1, v_2) \quad (17)$$

where $X(v_1, v_2)$ represents the intensity of 2DCOS, $v_1$ and $v_2$ represent two independent spectral variables, and $\emptyset(v_1, v_2)$ is the intensity of the two-dimensional correlated synchronization, and $\psi(v_1, v_2)$ is the strength of the two-dimensional correlation asynchrony (Zhang et al., 2015).

Synchronous spectroscopy can represent the overall similarity or consistency between the intensity changes caused by two independent optical variables and LPC disturbances.

# S2.3.3 PROSAIL-5B model

The version of the PROSAIL model used in this study is PROSAIL-5B (Jacquemoud et al., 2009), which is a coupling of the PROSPECT-5B model and the 4SAIL model: the former is a leaf optical model that simulates the reflectance and transmittance of leaves at 400-2500 nm; The latter is the canopy bidirectional reflectance model, which is improved to obtain vegetation canopy reflectance.

PROSAIL-5B is an effective way to simulate the inversion of maize leaf biomass from hyperspectral data. However, there are problems such as high input parameter uncertainty, difficulty in parameter tuning, pathological inversion, and slow speed in the inversion process. The parameter calibration of the model can obtain parameter values within the range of observed reflectance and uncertainty, provide rich and accurate parameter information, and reduce bias in the model inversion process. For model parameter calibration, the traditional method is to use trial and error or set empirical values, and the range of parameter values set is relatively rough, making it difficult to express the spatiotemporal differences of crop parameters in the study area. In addition, different parameters in the model may lead to the "same effect of different parameters" phenomenon of the same simulation result. In the inversion calculation, parameters that do not conform to the actual situation are taken as the calculation results, resulting in a large deviation between the inversion results and the measured values.

The PROSAIL model has numerous input parameters, including 13 parameters such as leaf structure parameters ($N_s$), leaf area index (LAI), and average leaf inclination angle (ALA). However, due to objective constraints, it is not possible to obtain specific values of vegetation parameters for all plots. Therefore, it is necessary to determine the coverage range of each parameter (Table S1), determine the magnitude of the impact of input parameters on the model simulation results through EFAST sensitivity analysis (Qiao et al., 2020), and determine highly sensitive parameters. Using the Mixing Sine and Cosine Algorithm with Lévy Flying Chaotic Sparrow Algorithm to optimize model parameters, determine the range of high sensitivity parameters and the values of other low sensitivity parameters. Sensitivity analysis of each parameter can better determine the type and number of parameters, reduce computational complexity in the calculation process, and improve the accuracy of model inversion.

Based on these parameters, simulate the leaf biomass of maize plants ($AGB_{Leaf}$) using leaf area index and dry matter content.

$$AGB_{Leaf}\left({}^{t}/_{ha}\right) = LAI \times Cm \times 4100 \times 15 \div 100 \tag{18}$$

Among them, one hectare is set as 15 acres, and one acre contains 4100 corn plants.

# S2.3.4 EFAST sensitivity analysis

Using the EFAST method to conduct sensitivity analysis of crop model parameters, and then localizing the calibration of the model. This includes local sensitivity analysis and global sensitivity

analysis. Local sensitivity analysis is the study of the impact of a single input parameter changing within a local range on the output response of a model. The method of local sensitivity analysis is simple and computationally intensive, but its disadvantage is that it cannot fully describe the spatial distribution of model parameters and ignores the interaction between parameters. Global sensitivity analysis can analyze the impact of the entire parameter space on model results, considering the interactions between parameters.

The EFAST method is a sensitivity analysis method based on variance decomposition proposed by Saltelli combining the advantages of Sobol' method (Sobol, 1993) and Fourier amplitude sensitivity test (R. et al., 1977). It can calculate the contribution rate of each parameter and its interaction to the variance of the model results. The basic idea of this method comes from Bayesian theorem, which states that the sensitivity of the output results of a pattern can be reflected by the variance of the pattern results. The sensitivity of parameter $x$ can be represented by the following equation:

$$Sensitivity_x = \frac{var_x[E(Y|X)]}{var(Y)} \tag{19}$$

wherein, $Y$ is the output value of the pattern, $x$ is the input parameter, E(Y|X) is the expected value of $Y$ when $x$ takes a certain value, and $var_x$ is the variance when $x$ traverses the range of values.

According to the Sobol' method of total variance decomposition, the total variance output by the model can be decomposed into the sum of the variances of each parameter and the variances of parameter interactions, and sensitivity quantification can be performed by quantifying the contribution ratio of parameters to the output variance.

Step1: Convert the model $y = f(x_1, x_2, ..., x_n)$ to $y = f(s)$.

$$x_i = \frac{1}{2} + \frac{\arcsin\left[\sin\left(\omega_i s + \varphi_i\right)\right]}{\pi}, s \in [-\infty, +\infty], i = 1,2,\cdots,n \tag{20}$$

Step2: Expand $f(s)$ using Fourier series:

$$y = f(s) = \sum_{j=-\infty}^{+\infty} \left[A_j \cos(js) + B_j \sin(js)\right] \tag{21}$$

wherein, $A_j$ and $B_j$ are Fourier coefficients.

$$A_j = \frac{1}{2\pi} \int_{-\pi}^{\pi} f(s) \cos(js)\, ds \tag{22}$$

$$B_j = \frac{1}{2\pi} \int_{-\pi}^{\pi} f(s) \sin(js)\, ds \tag{23}$$

Step3: Calculate total variance:

$$var(y) = \frac{1}{2\pi} \int_{-\pi}^{\pi} f^2(s) ds - \left[\frac{1}{2\pi} \int_{-\pi}^{\pi} f(s)\, ds\right]^2 \tag{24}$$

Step4: Calculate the first-order influence index $S_i$, which is the sensitivity of parameter $i$:

$$S_i = \frac{var_i(y)}{var(y)} \tag{25}$$

Step5: Calculate the total effect index $S_{Ti}$, which is the sensitivity of the main effects and interactions of parameter $i$:

$$S_{Ti} = 1 - \frac{var_{\sim i}(y)}{var(y)} \tag{26}$$

wherein, $var_{\sim i}(y)$ is the conditional variance estimate except for parameter $i$.

# S2.3.5 Mixing Sine and Cosine Algorithm with Lévy Flying Chaotic Sparrow Algorithm

Assuming there are n sparrows in the population, the population composed of all individuals is $X = [x_1, x_2, \cdots, x_n]^T$.

$$X = \begin{bmatrix} x_{1,1} & x_{1,2} & \cdots & x_{1,d} \\ x_{2,1} & x_{2,2} & \cdots & x_{2,d} \\ \vdots & & \vdots & \vdots \\ x_{n,1} & x_{n,2} & \cdots & x_{n,d} \end{bmatrix} \tag{27}$$

The fitness functions for each individual are $\mathrm{F} = [f(x_1), f(x_2), \cdots, f(x_n)]^T$.

$$F = \begin{bmatrix} f([x_{1,1} & x_{1,2} & \cdots & x_{1,d}]) \\ f([x_{2,1} & x_{2,2} & \cdots & x_{2,d}]) \\ \vdots & & \vdots & \vdots \\ f([x_{n,1} & x_{n,2} & \cdots & x_{n,d}]) \end{bmatrix} \tag{28}$$

$$X_{i,j}^{t+1} = \begin{cases} X_{i,j}^t \cdot \exp\left(\dfrac{-i}{\alpha \times iter_{max}}\right), R_2 < ST \\ X_{i,j}^t + Q \cdot L, \qquad R_2 \geq ST \end{cases} \tag{29}$$

wherein, *t*: Current iteration count; $X_{i,j}^t$: the position of the *i*-th sparrow in the *j*-th dimension in the *t*-th generation; $\alpha \in (0,1)$; $Iter_{max}$: Maximum number of iterations; $R_2$: Alarm value; ST: Safety threshold; Q: Random numbers that follow a normal distribution; L: 1-dimensional all 1 matrix.

$$X_{i,j}^{t+1} = \begin{cases} Q \cdot \exp\left(\dfrac{X_{worst}^t - X_{i,j}^t}{i^2}\right),\ i > \dfrac{n}{2} \\ X_P^{t+1} + \left|X_{i,j}^t - X_P^{t+1}\right| \cdot A^+ \cdot L,\ i \leq \dfrac{n}{2} \end{cases} \tag{30}$$

wherein, $X_{worst}^t$: The position of the individual with the worst fitness in the *t*-th generation; $X_P^{t+1}$: Represents the position of the individual with the best fitness in the *t*+1 generation; A: A one-dimensional matrix, with each element randomly preset to -1 or 1; $A^+=A^T(AA^T)^{-1}$.

$$X_{i,j}^{t+1} = \begin{cases} X_{best}^t + \beta \cdot \left|X_{i,j}^t - X_{best}^t\right|, f_i \neq f_g \\ X_{best}^t + k \cdot \left(\dfrac{X_{i,j}^t - X_{best}^t}{|f_i - f_w| + \varepsilon}\right), f_i = f_g \end{cases} \tag{31}$$

wherein, $X_{best}^t$: the global optimal position in the *t*-th generation; $\beta$: Step size follows a normal distribution with a mean of 0 and a variance of 1; $k \in [-1,1]$; Set $\varepsilon$ as a constant to avoid having a denominator of 0; $f_i$: The fitness value of the current individual; $f_g$: The current fitness value of the globally optimal individual; $f_w$: The current global worst individual fitness value.

$$\omega = \omega_{min} + (\omega_{max} - \omega_{min}) \cdot \sin\left(\frac{t\pi}{iter_{max}}\right) \tag{32}$$

$$X_{i,j}^{t+1} = \begin{cases} (1-\omega) \cdot X_{i,j}^t + \omega \cdot \sin(r_1) \cdot \left|r_2 \cdot X_{best} - X_{i,j}^t\right|, R_2 < ST \\ (1-\omega) \cdot X_{i,j}^t + \omega \cdot \cos(r_1) \cdot \left|r_2 \cdot X_{best} - X_{i,j}^t\right|, R_2 \geq ST \end{cases} \tag{33}$$

wherein, $r_1$ is a random number within [0, 2π] , and $r_2$ is a random number within [0, 2].

$$X_{i,j}^{t+1} = \begin{cases} Q \cdot \exp\left(\frac{X_{worst}^{t} - X_{i,j}^{t}}{i^2}\right), \ i > \frac{n}{2} \\ X_P^{t+1} + X_P^{t+1} \oplus Levy(d), \ i \le \frac{n}{2} \end{cases} \quad (34)$$

## S2.3.6 Modelling evaluation

Evaluate the calibration model by comparing the coefficient of determination ($R^2$) and root mean square error (RMSE). The ratio of standard deviation (SD) to root mean square error (RMSE) is called RPD, which is used to evaluate the predictive accuracy of a model. The model is divided into four levels: 1) poor prediction (RPD<1.5), 2) moderate prediction (1.5≤RPD<2.0), 3) approximate prediction (2.0 ≤ RPD<3.0), and 4) good prediction (RPD ≥ 3.0).

The values of $R^2$, RMSE, SD, and RPD are calculated using the following equation:

$$R^2 = \frac{\sum_{n=1}^{N}(\hat{y}_n - \bar{y})^2}{\sum_{n=1}^{N}(y_n - \bar{y})^2} \quad (35)$$

$$RMSE = \sqrt{\frac{\sum_{n=1}^{N}(y_n - \hat{y}_n)}{N}} \quad (36)$$

$$nRMSE[\%] = \frac{RMSE}{\text{measured value range}} \times 100 \quad (37)$$

$$RPD = \frac{SD}{RMSE} \quad (38)$$

where, $\hat{y}_n$ and $y_n$ are predicted and measured P, respectively; *N* is the sample number in the calibration, validation dataset; *n* is the sequence number.

# S2.4 Regression algorithm

## S2.4.1 Support Vactor Regression

Support Vector Regression (SVR) is a machine learning method based on structural risk minimization (SRM) to solve nonlinear regression problems. The core principle of SVR is to fit data by minimizing prediction errors and maintaining a boundary (interval) during the fitting process, so that most data points fall within this boundary.

## S2.4.2 Random Forest Regression

Random Forest (RF) regression is an ensemble learning based algorithm that performs regression tasks by constructing multiple decision trees and integrating their prediction results. In the process of generating numerous decision trees, random sampling is performed on the sample observations and feature variables of the modeling dataset. Each sampling result is a tree, and each tree generates rules and judgment values that match its own attributes. The forest ultimately

integrates the rules and judgment values of all decision trees to achieve the regression of the random forest algorithm.

### S2.4.3 Extreme Learning Machines Regression

Extreme Learning Machine (ELM) is a single hidden layer feedforward neural network algorithm, whose model structure consists of three layers: input layer, hidden layer, and output layer. Similar to artificial neural networks, the connections between each layer are completed using feature mapping functions. The information in the input layer is processed and transmitted to the output layer through the hidden layer, and then the output layer calculates the value based on the mapping function.

## S3. Results

## S3.2.2 Empirical model prediction of shoot P uptake

According to the photochemical properties, the spectral band of 350-2500 nm is divided into different intervals: the Violet Blue band (VB: 350-449 nm); Blue band (B: 450-510 nm); Blue Edge (BE: 490-530 nm); Green band (G: 530-590 nm); Yellow Edge (YE: 560-640 nm); Red band (R: 640-670 nm); Red Edge (RE: 680-760 nm); Near-Infrared-1 band (NIR1: 760-860 nm); Near-Infrared-2 band (NIR2: 861-1040 nm); Short-Wave-Infrared-1 band (SWIR1: 1040-1350 nm); Short-Wave-Infrared-2 band (SWIR2: 1550-1850 nm); Short-Wave-Infrared-3 band (SWIR3: 2080-2350 nm) for evaluating spectral sensitivity changes at different growth stages.

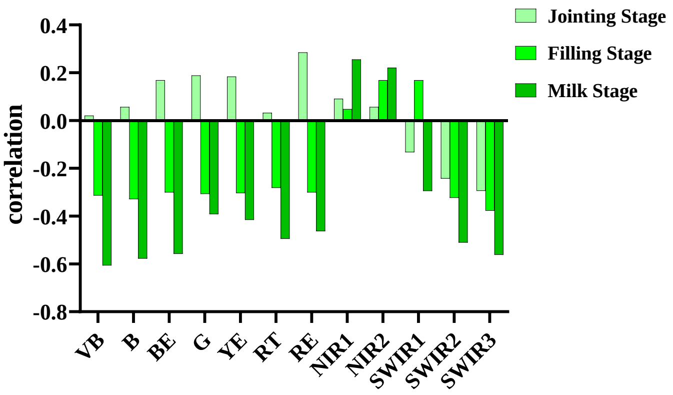


**Fig. S1** Correlation between AGB of maize and jointing, filling, and milk stages.

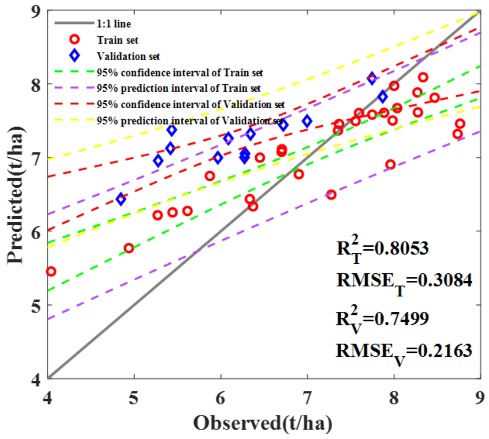


**Fig.S2** Random Forest model based on NDSI.

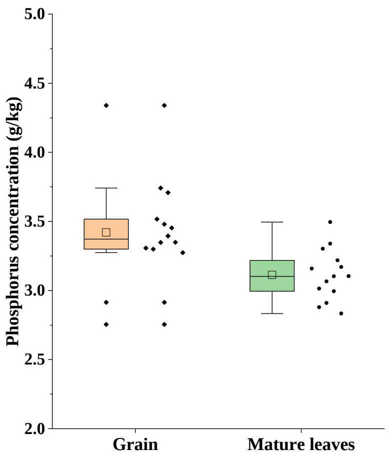


**Fig. S3** Distribution of P content in mature leaves and grains.

# S3.3.1 EFAST sensitivity analysis

**Table S1.** Spectral sensitivity changes at different growth stages.

| Parameter | Sensitive band | |
|---|---|---|
| | Filling period | milk ripening period |
| VB | 444nm | 428nm |
| B | 472nm | 450nm |
| BE | 490nm | 490nm |
| G | 555nm | 530nm |
| YE | 560nm | 640nm |
| RT | 670nm | 670nm |
| RE | 680nm | 680nm |
| NIR1 | 786nm | 786nm |

| | | |
|---|---|---|
| **NIR2** | 1039nm | 861nm |
| **SWIR1** | 1041nm | 1350nm |
| **SWIR2** | 1806nm | 1835nm |
| **SWIR3** | 2281nm | 2345nm |

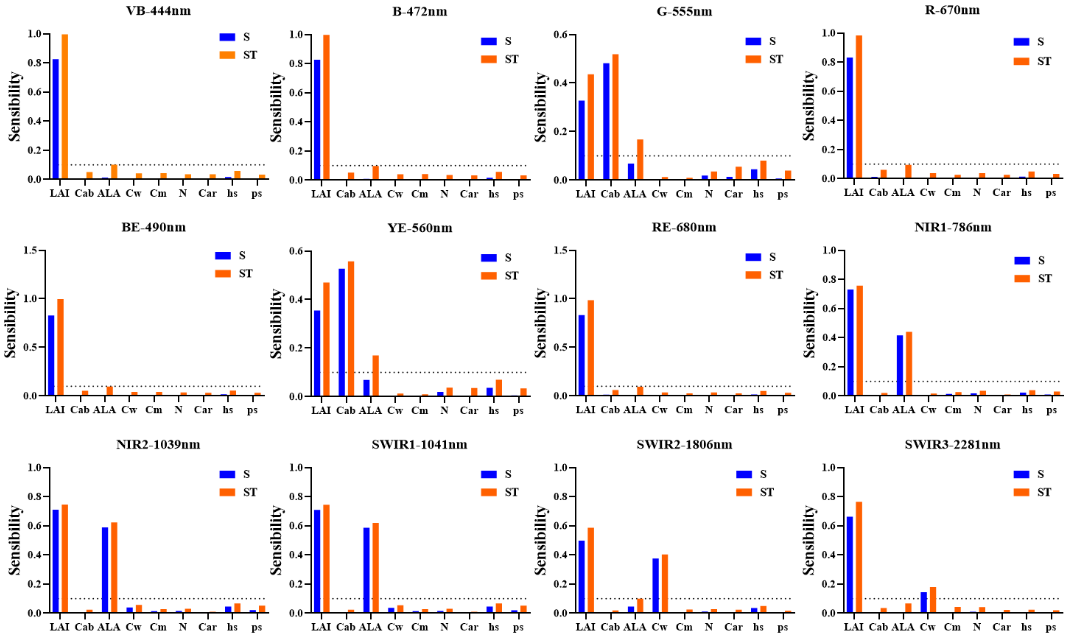


**Fig.S4** Sensitivity analysis results during Grain-filling period.

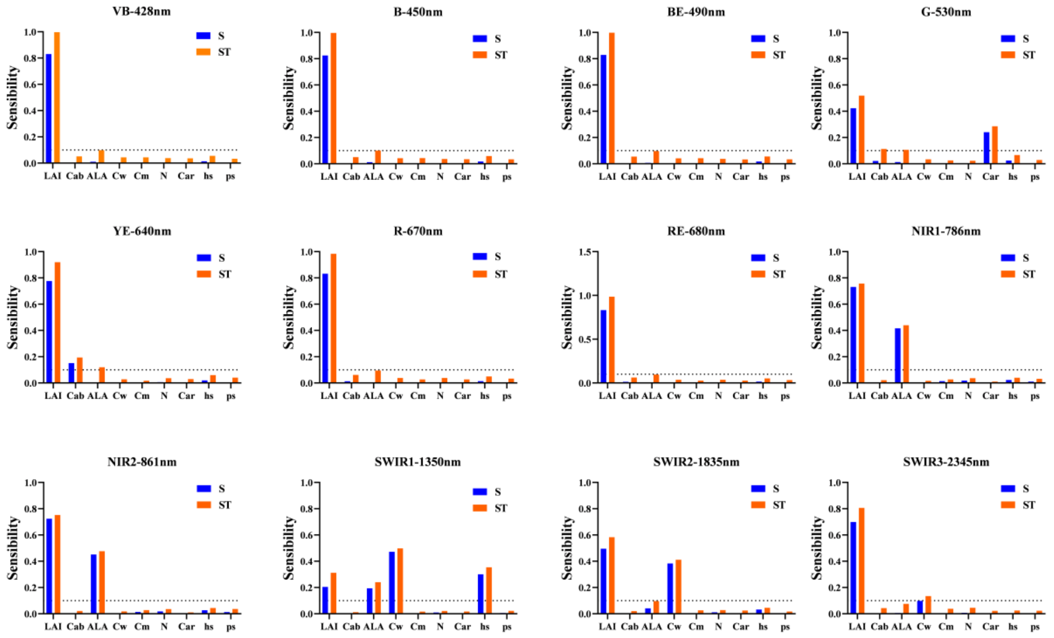


**Fig.S5** Sensitivity analysis results during milk stage.

**Table S2.** Parameter values of PROSAIL model.

| Symbol | Parameter | Range | Std. | References |
|---|---|---|---|---|
| | | **Leaf Model: (PROSPECT-D)** | | |

| | | | | |
|---|---|---|---|---|
| **N** | Leaf structure index | 1.2-1.8 | 1.8 | Chai et al. (2021) |
| **Cab** | Leaf chlorophyll content | 20-70 | 18-22<br>(Step size: 1) | Guo et al. (2023) |
| **Car** | Total carotenoid content | 4-15 | 3.5-4.5<br>(Step size: 0.5) | Huang et al. (2023)<br>Chai et al. (2021) |
| **Cbrown** | Brown pigments | 0 | 0 | Huang et al. (2023) |
| **Cw** | Leaf water content | 0.01-0.05 | 0.01-0.02<br>(Step size: 0.004) | Guo et al. (2023) |
| **Cm** | Dry matter content | 0.004-0.0075 | 0.004-0.005<br>(Step size: 0.0001) | Guo et al. (2023) |
| | | **Canopy Model: (4SAIL)** | | |
| **LAI** | Leaf area index | 0.5-7 | 0.5-1.2<br>(Step size: 0.05) | Kayad et al. (2022)<br>Nie et al. (2023) |
| **ALA** | Average leaf angle | 20-70 | 20-21<br>(Step size: 1) | Koetz et al. (2005) |
| **hspot** | Hot-spot parameter | 0.001-0.450 | 0.25-0.4<br>(Step size: 0.05) | Sun et al. (2021)<br>Su et al. (2019) |
| **psoil** | Soil brightness | 0.4-0.7<br>(Filling period)<br>0.8-1<br>(Milk period) | 0.655 | Nie et al. (2023) |
| **tts** | Solar zenith angle | \ | 30 | |
| **tto** | Observation zenith angle | \ | 0 | Sun et al. (2021) |
| **psi** | Azimuth | \ | 0 | |

*Range: Range in literatures; Std.: Optimized standard value.

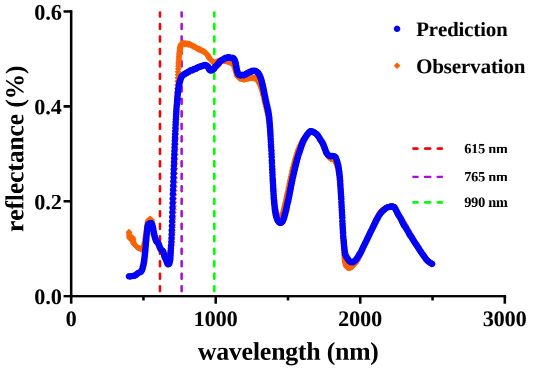


**Fig.S6** Comparison between simulated spectra and measured spectra.

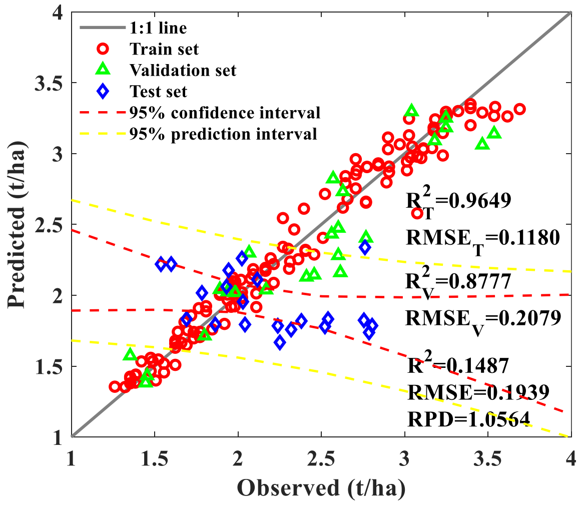


**Fig.S7** Leaf biomass prediction model based on practical constraints.

# S4. Discussion

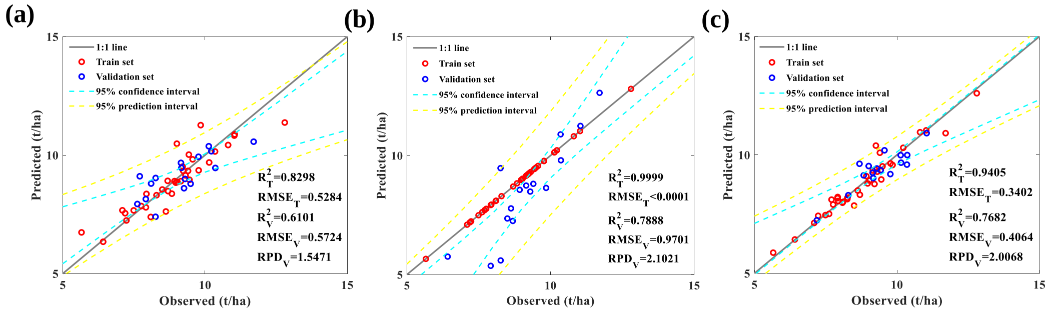


**Fig.S8** Prediction models based on sensitive bands for the (a) original spectrum, (b) first-order differential spectrum, and (c) second-order differential spectrum.

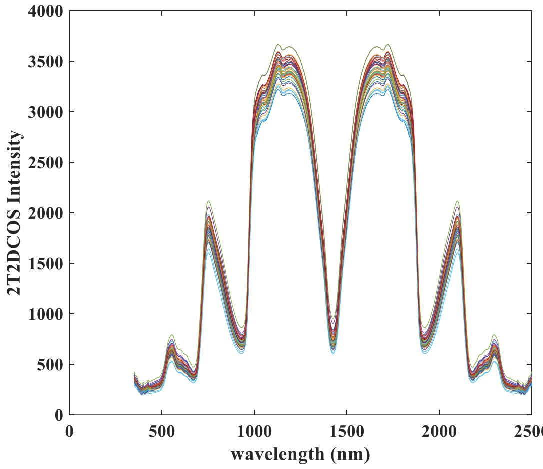


**Fig. S9** Autocorrelation peak extracted from the filling-milk-2T2DCOS synchronous spectrum.

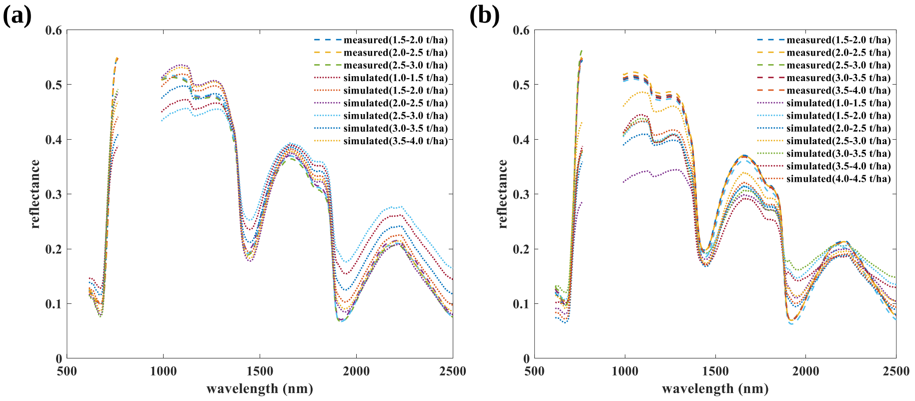


**Fig. S10** Average simulated spectra within different leaf biomass range under (a) real range constraints and (b) NRBO-AL constraints.

# References

CHAI, L. N., JIANG, H. Y., CROW, W. T., LIU, S. M., ZHAO, S. J., LIU, J. & YANG, S. Q. 2021. Estimating Corn Canopy Water Content From Normalized Difference Water Index (NDWI): An Optimized NDWI-Based Scheme and Its Feasibility for Retrieving Corn VWC. *Ieee Transactions on Geoscience and Remote Sensing,* 59**,** 8168-8181.

GUO, A. T., YE, H. C., HUANG, W. J., QIAN, B. X., WANG, J. J., LAN, Y. B. & WANG, S. Z. 2023. Inversion of maize leaf area index from UAV hyperspectral and multispectral imagery. *Computers and Electronics in Agriculture,* 212**,** 14.

HUANG, X., GUAN, H. D., BO, L. Y., XU, Z. Q. & MAO, X. M. 2023. Hyperspectral proximal sensing of leaf chlorophyll content of spring maize based on a hybrid of physically based modelling and ensemble stacking. *Computers and Electronics in Agriculture,* 208**,** 15.

JACQUEMOUD, S., VERHOEF, W., BARET, F., BACOUR, C., ZARCO-TEJADA, P. J., ASNER, G. P., FRANçOIS, C. & USTIN, S. L. 2009. PROSPECT plus SAIL models: A review of use for vegetation characterization. *Remote Sensing of Environment,* 113**,** S56-S66.

KAYAD, A., RODRIGUES, F. A., NARANJO, S., SOZZI, M., PIROTTI, F., MARINELLO, F., SCHULTHESS, U., DEFOURNY, P., GERARD, B. & WEISS, M. 2022. Radiative transfer model inversion using high-resolution hyperspectral airborne imagery - Retrieving maize LAI to access biomass and grain yield. *Field Crops Research,* 282**,** 12.

KOETZ, B., BARET, F., POILVé, H. & HILL, J. 2005. Use of coupled canopy structure dynamic and radiative transfer models to estimate biophysical canopy characteristics. *Remote Sensing of Environment,* 95**,** 115-124.

NIE, C. W., SHI, L., LI, Z. H., XU, X. B., YIN, D. M., LI, S. K. & JIN, X. L. 2023. A comparison of methods to estimate leaf area index using either crop-specific or generic proximal hyperspectral datasets. *European Journal of Agronomy,* 142**,** 11.

NODA 1993. Generalized Two-Dimensional Correlation Method Applicable to Infrared, Raman, and Other Types of Spectroscopy. *Applied Spectroscopy*.

NODA, I. 2018. Two-trace two-dimensional (2T2D) correlation spectroscopy - A method for extracting useful information from a pair of spectra. *Journal of Molecular Structure***,** 471-478.

QIAO, K., ZHU, W. Q. & XIE, Z. Y. 2020. Application conditions and impact factors for various vegetation indices in constructing the LAI seasonal trajectory over different vegetation types. *Ecological Indicators,* 112.

R., I., CUKIERT, K. & SHULER 1977. Nonlinear sensitivity analysis of multiparameter model systems. *The Journal of Physical Chemistry,* 81**,** 2365-2366.

SOBOL, I. M. Sensitivity Estimates for Nonlinear Mathematical Models. 1993.

SOWMYA, R., PREMKUMAR, M. & JANGIR, P. 2024. Newton-Raphson-based optimizer: A new population-based metaheuristic algorithm for continuous optimization problems. *Engineering Applications of Artificial Intelligence,* 128.

SU, W., ZHANG, M. Z., BIAN, D. H., LIU, Z., HUANG, J. X., WANG, W., WU, J. Y. & GUO, H. 2019. Phenotyping of Corn Plants Using Unmanned Aerial Vehicle (UAV) Images. *Remote Sensing,* 11**,** 19.

SUN, B., WANG, C. F., YANG, C. H., XU, B. D., ZHOU, G. S., LI, X. Y., XIE, J., XU, S. J., LIU, B., XIE, T. J., KUAI, J. & ZHANG, J. 2021. Retrieval of rapeseed leaf area index using the PROSAIL model with canopy coverage derived from UAV images as a correction parameter.

*International Journal of Applied Earth Observation and Geoinformation,* 102**,** 10.

ZHANG, Y., ZHENG, L. H., LI, M. Z., DENG, X. L. & JI, R. H. 2015. Predicting apple sugar content based on spectral characteristics of apple tree leaf in different phenological phases. *Computers and Electronics in Agriculture,* 112**,** 20-27.
